# Transfer potential of F-like plasmids in *Escherichia coli* differs by animal environment

**DOI:** 10.1093/ismeco/ycag256

**Published:** 2026-09-06

**Authors:** Sneha Sundar, Sebastian Bonhoeffer, Jana S Huisman

**Affiliations:** Institute of Integrative Biology, ETH Zürich, Universitätstrasse 16, CH-8092 Zürich, Switzerland; Institute of Integrative Biology, ETH Zürich, Universitätstrasse 16, CH-8092 Zürich, Switzerland; Physics of Living Systems, Massachusetts Institute of Technology, 77 Massachusetts Ave, Bldg. 4-332, Cambridge, MA 02139, USA

**Keywords:** mobile genetic element (MGE), E. coli, horizontal gene transfer (HGT), conjugation, One Health, genomic epidemiology, plasmid identification, ecology

## Abstract

Plasmids play a key role in the spread of virulence and antimicrobial resistance genes to new genetic backgrounds. Genetic variation in the transfer operon, which contains the genes responsible for conjugation, can lead to substantial differences in transfer potential even between closely-related plasmids. However, it is not clear how much genetic diversity there is in transfer operons of natural bacterial populations. Here, we analyse the prevalence and transfer potential of F-like plasmids, a clinically important family of plasmids in *Enterobacteriaceae*, by searching for the presence of essential conjugation genes in 1200 *Escherichia coli* genomes isolated from three livestock-associated environments. We find that the fraction of functionally complete F-like transfer operons was significantly higher in poultry than in bovine- and swine-associated bacteria. This difference was not captured in methods that use the presence of replication genes to estimate plasmid prevalence. Confounders such as the phylogenetic relatedness of *E. coli* or the presence of antibiotic resistance could not explain these significant differences in transfer potential. Instead, it seems the poultry environment selects for plasmids with high transfer potential, as it also contained more conjugative plasmid types per isolate. While we find environment-specific differences in overall plasmid frequency, patterns of transfer gene presence/absence were similar across the three environments. Regulatory and exclusion genes are the exception to this pattern, suggesting environment-specific modulation of transfer rates. This highlights the use of genomic data to uncover environment-specific differences in plasmid prevalence and transfer potential, revealing the selection pressures shaping horizontal gene transfer in these environments.

## Introduction

Plasmids are key vectors in the spread of clinically important traits such as antibiotic resistance, virulence, and metabolic genes to new genetic backgrounds [1–5]. A plasmid’s ability to transfer is contingent on the presence of functional transfer genes (*tra* genes), which encode and regulate the structures necessary for conjugation [6]. For a few model plasmids the mechanistic basis of conjugation has been studied extensively *in vitro* [7, 8], yet little is known about the genetic diversity and function of transfer genes in natural environments. This limits our ability to predict how plasmids spread in natural bacterial populations, and whether there are any differences depending on the ecological context.

The genetic diversity of plasmids is shaped by environment-specific evolutionary selection pressures. Specifically, a plasmid must balance the growth cost it places on the bacterial host with horizontal spread and selection for plasmid-borne genes to optimize their reproductive fitness in a given environment [9–11]. Conjugative plasmid carriage and the process of conjugation impose costs on the host cell [10, 12]. In experimental settings, these costs are compensated over time by bacterial host or plasmid mutations [12–14] or even the loss of transfer genes [15]. Phages that target conjugative pili for entry also select for plasmids that lack functional conjugation machinery [16] and such phages are abundant in natural samples [17].

These selection pressures may alter a plasmid’s ability to transfer, i.e. its transfer potential. Experiments have shown that a loss of structural genes involved in forming the type IV secretion system (T4SS) renders a plasmid incapable of conjugating on its own [7, 8]. Loss of regulatory genes has been reported to affect conjugation efficiency [18, 19], while loss of exclusion genes may increase superinfection of closely-related plasmids in the same host cell [20, 21]. Depending on the genes that are lost, the plasmid may still be able to transfer if another plasmid with a functional conjugation system is present (termed mobilizable plasmid), or be rendered completely immobile (termed non-mobilizable plasmid). In natural samples, mobilizable plasmids are common, representing 21% of all plasmids [22].

Plasmids are thought to transition from conjugative to mobilizable states frequently [22, 23], and a diversity of transfer operon structures exists among closely-related plasmids with plasmids often missing one or more transfer genes [24, 25]. Since the genomic presence/absence of *tra* genes has been shown to be a good determinant of transfer potential in experiments with natural IncF and IncI plasmids [26], these genes may serve as useful functional markers to estimate both plasmid prevalence and transfer potential.

Existing studies that investigate natural plasmid diversity in relation to mobility usually suffer from one of three issues. First, they rely on sequence databases, which may represent a biased sample of plasmid diversity: clinically relevant and experimentally used plasmids tend to be overrepresented, and the plasmids do not necessarily represent the plasmid populations found in a well-defined ecological niche [22, 24, 27–29]. Second, plasmid prevalence is often assessed based solely on the replicon sequence [30–33]. While the replicon is useful to define incompatibility groups, it is less informative about transfer potential. Third, methods designed to assess plasmid mobility typically use very relaxed thresholds to assess transfer potential. Existing mobility typing schemes have either used (i) just the relaxase, which only distinguishes mobilizable plasmids from non-mobilizable ones [34, 35], or (ii) the relaxase and a small number of other conjugation-related genes [22, 36, 37]. The latter approach can distinguish conjugative, mobilizable, and non-mobilizable plasmids. Yet in practice, the thresholds used to classify a plasmid as conjugative are not specific to the conjugation system assessed and are lacking a functional basis. For instance, bioinformatic studies on plasmid mobility have used as little as three mating pair formation (MPF) system genes to call F-like plasmids conjugative [22, 36], while experiments have demonstrated that >10 MPF genes are required for conjugation [6–8].

Here, we study the prevalence, genetic diversity, and transfer potential of F-like plasmids in natural *Escherichia coli* (*E. coli*) populations. We analyse a collection of 1200 *E. coli* genomes in three livestock-associated environments (swine, poultry, and cattle) for the presence of 35 F-like transfer genes (Fig. 1A). We define a new metric of transfer operon completeness, which is functionally linked to transfer potential, and find notable differences in plasmid prevalence and completeness across the three livestock-associated environments. This has important epidemiological implications for the spread of plasmid-borne resistance in *Enterobacteriaceae*. Our work also highlights the benefits of integrating molecular insights into bioinformatic analyses, to elucidate evolutionary selection pressures influencing plasmid spread in natural environments.

**Figure 1:**
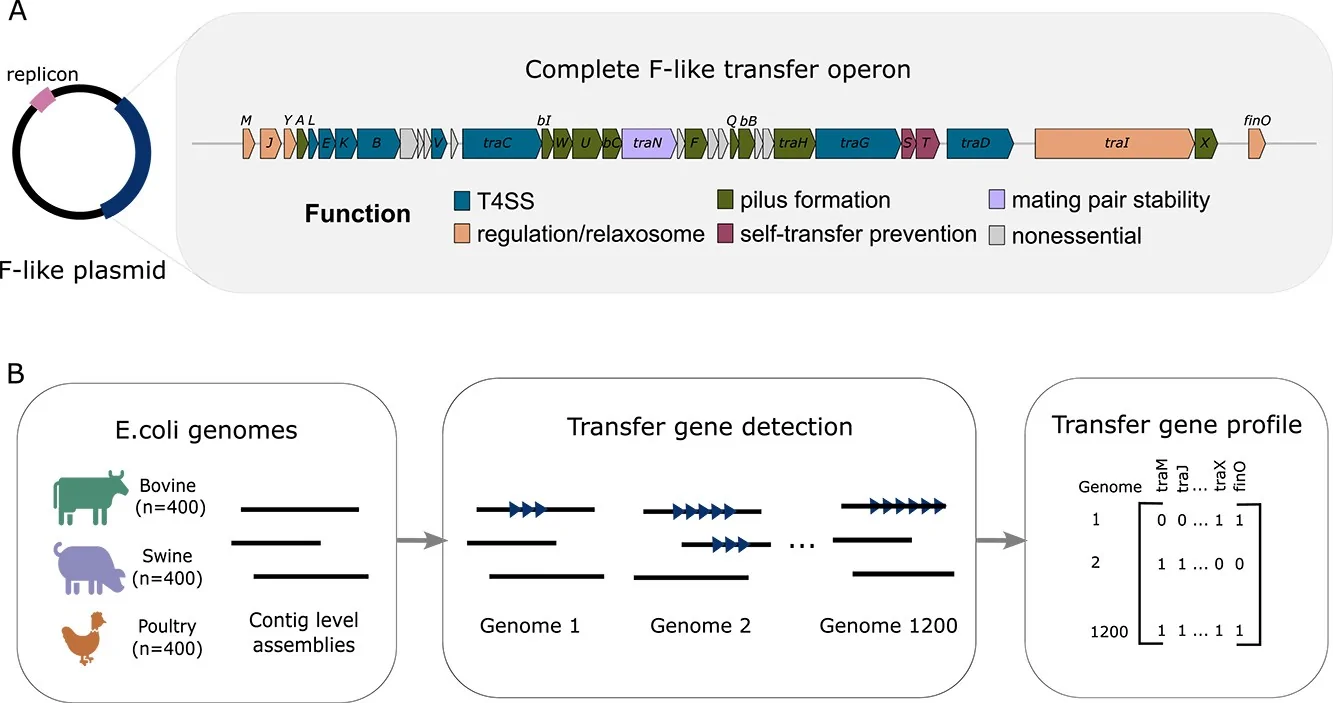
**A.** Schematic of the F-like plasmid with a replicon and an F-like transfer operon. A magnified view of the operon displays individual genes, where colours indicate the function of different genes in the operon. **B.** Bioinformatic pipeline to detect transfer genes: (i) we collected 1200 contig-level genome assemblies associated with three animal environments, (ii) used a combined sequence homology and annotation-based approach to identify a set of 35 genes from the F plasmid transfer operon, and (iii) determined a presence/absence profile of F-like transfer genes for each genome.

## Material and methods

### *Escherichia coli* dataset

We used a collection of 1200 publicly available *E. coli* genomes associated with livestock, sampled as part of routine surveillance from USA between years 2010 and 2018 (Fig. S1). The genomes used were not preferentially isolated based on virulence, resistance, or strain characteristics. Our collection represents a subset of the dataset used in [38], randomly subsampled to select equal numbers (*n=400* each) of bovine, swine, and poultry-associated genomes, corresponding to the number of swine-associated samples available. The genomes had all been processed with a common assembly pipeline from Enterobase to generate contig-level assemblies and passed Enterobase’s internal assembly quality standards [39, 40], which include *E. coli*-specific criteria for the number of bases in the assembly, N50 value, number of contigs, proportion of scaffolding placeholders, and species assignment [40]. The quality of the draft genomes were also independently assessed using checkM2 (v.1.1.0, using default settings) [41] and were found to be high quality (*≥* 99.9% completeness, <3% contamination). Details on the metadata associated with the genomes—phylogroup, antimicrobial resistance gene (ARG) presence, sample source, and sampling year—can be found in the Supplementary Text.

### Detection of F-like transfer genes from short-read bacterial assemblies

To identify F-like transfer genes, we first used a sequence similarity based gene finding approach using a reference database of transfer gene sequences from *Enterobacteriaceae* F-like plasmids adapted from Fernandez-Lopez *et al.* [24] (see Supplementary Text). This reference dataset was comprised of sequence homologues of 35 F-like *tra* genes (Table S1).

Using this set of *tra* homologues as queries, we ran tblastn (BLAST+ 2.12.0) [42] to identify F-like transfer operon associated contigs in the *E. coli* genomes. To allow for distant homologues, we selected hits with a minimum percent identity (pid) of 50%. To allow partial genes or genes on the edge of contigs to be detected, we selected a minimum query coverage of 30%. The best hit for each of the 35 *tra* marker genes (defined as the hit with the highest bitscore, pid, and query coverage, prioritized in this order) was selected from each contig. In this way, contigs containing one or more F-like transfer genes were identified as likely F-like plasmid-associated contigs. We found an excess of contigs with only the transfer gene *traT*. As chromosomal homologues of *traT* have been reported before [43], we inspected the other genes on these contigs and found them to be chromosome-associated. We filtered out such contigs with *traT* as the only *tra* gene.

We then used the bacterial genome and plasmid annotation tool Bakta (v. 1.8.1, with default settings) [44] to independently cross-check the hits found by our *tra* gene-specific BLAST. We used the annotations shown in Table S1 to identify F-like transfer genes. Overall, we found little difference between the Bakta and BLAST approach in *tra* gene presence/absence. However, Bakta was better in identifying the entry exclusion gene *traS*, which has been described to have low sequence homology [24, 45]. For this reason, we used the set of transfer genes identified by Bakta in subsequent analysis.

### Measuring prevalence of F-like transfer operons, replicons, and transfer genes

An F-like transfer operon was determined as present if more than a given number of F-like transfer genes (the detection threshold) were detected in the genome. Prevalence of F-like transfer operons, defined as the ratio of the number of genomes containing an F-like transfer operon to the total number of genomes assessed, was evaluated for a range of detection thresholds (1–35 genes).

Replicon sequences in each genome were detected using the tool PlasmidFinder (v. 2.1.6) [46] with default parameters (minimum identity 95% and minimum coverage 60%). PlasmidFinder detects plasmids in genomes using a predefined database of replicon sequences as queries in a BLAST search. The genome was said to contain an F-like replicon if one or more replicons of type IncFIA, IncFIB, IncFIC, or IncFII were detected. Prevalence of F-like replicons was defined as the number of genomes containing an F-like replicon divided by the total number of genomes assessed.

We estimated transfer gene frequencies using a gene content matrix with 1200 isolates (rows) and 35 transfer genes (columns), where each entry represents the number of detected hits for a given gene in an isolate. A binary presence–absence matrix was derived from this matrix (1 = present, 0 = absent). Gene frequencies were calculated as the proportion of genomes in which each gene was detected, conditioned on genomes containing at least one transfer gene (*n=249* in bovine, *n=350* in poultry, *n=273* in swine) to control for differences in the proportion of genomes lacking transfer genes across animal sources.

### Classifying transfer potential of F-like transfer operons

We used molecular insights from experimental studies to classify the transfer potential of F-like plasmids based on the presence/absence of a set of 23 essential transfer genes (Table S1 and Supplementary Text).

Every genome was classified as containing a ‘complete’, ‘incomplete’, or ‘missing’ F-like transfer operon depending on the number of essential genes detected in the genome. Genomes were classified as ‘missing’ F-like transfer operons if less than five genes from the essential gene set were detected, as ‘incomplete’ if *≥*5 but <23 genes were detected, and ‘complete’ if all 23 essential genes were detected.

### Logistic regression model

The presence of a complete F-like operon was modelled using logistic regression implemented as a generalized linear model with a binomial error distribution using the function *glm* in R version 4.2.2. Candidate explanatory variables included Animal Environment (poultry, bovine, swine), Phylogroup (A–F), Sample Source (food, livestock), antibiotic resistance gene (ARG) presence (yes,no), and sampling year (before 2017, 2017, or later).

Model selection was performed using Akaike’s Information Criterion (AIC). All combinations of explanatory variables were tested to identify the main-effects model with the lowest AIC (Table S4). Pairwise interaction terms were then added sequentially to the selected main-effects model, and again the model with the lowest AIC was selected (Table S5).

The final selected model included Animal Environment, Phylogroup, Sample Source, and ARG presence as main effects, as well as an interaction between Phylogroup and ARG presence. Pairwise contrasts were computed using the *emmeans* package (v. 1.10.5) [47] with this selected model and multiple testing correction was performed using the Tukey method.

### Detecting non-F conjugation systems

We used geNOMAD (v. 1.7.4) [48] to identify the presence of non-F conjugation systems in our isolates. We computed two binary matrices (1 = present, 0 = absent) for MPF system type presence (1200 x 4) and relaxase type presence (1200 x 6). An MPF type is considered present if we could detect at least three distinct genes specific to that MPF type. This is analogous to how we defined F-like transfer operon presence but scaled to accommodate transfer systems with fewer MPF genes. A relaxase type is termed present in an isolate if we could detect at least one gene of that type in the isolate. Using these matrices, we computed the number of non-F MPF types per isolate and non-F relaxase types per isolate. The prevalence of each MPF type and relaxase type was also calculated from this binary matrix as the proportion of genomes in which an MPF or relaxase type was detected.

## Results

### F-like transfer operons are less prevalent than IncF replicons in *E. coli* from livestock

We set out to study the prevalence and genetic diversity of F-like plasmids in natural environments. To do so, we used a collection of 1200 publicly available *E. coli* genomes from the USA associated with three livestock environments [38]: bovine (*n = 400*), swine (*n = 400*), and poultry (*n = 400*). The isolates were sampled as part of routine farm surveillance by the USA Food and Drug Administration between 2010 and 2018 (Fig. S1) and were not specifically isolated based on any virulence, strain, or resistance characteristics. On top of passing assembly quality standards set by Enterobase [40], the quality of the draft genomes was independently assessed using checkM2 [41] and was found to be high quality (Fig. S2). All 1200 genomes had a completeness score *≥*99.9%, out of which 1194 genomes were complete (100% completeness score).

We assessed how similar the genomes were in terms of their pairwise average nucleotide identity (ANI). The ANI distribution follows the typical patterns expected for *E. coli* [49]: all pairs had ANI >96%, genomes belonging to the same PhG had average pairwise ANI of 99%, and the same ST 99.5% (Figs S3A,B). We found 42 clusters of highly similar genomes (121 genomes in total or 10% of the dataset) with ANI values *≥*99.99%, which meets a recommended ANI threshold for strain identification [50] (Fig. S3C). However, these potential strains could still have substantial differences in mobile genetic element (MGE) content [49, 51], which are not captured using ANI. Highly similar genomes may also be sampled because they are more prevalent. Therefore, for most analyses presented we do not perform any genome dereplication.

We developed a method using a combined sequence homology and annotation-based approach to detect a set of 35 genes from the F plasmid transfer operon in our *E. coli* genomes (Fig. 1). This resulted in a presence/absence profile of F-like transfer genes for each genome (Fig. 1B). Since we work with contig-level assemblies, plasmid sequences are likely to be fragmented across multiple contigs (Fig. S4 [52, 53]. We bypass this structural limitation by using a gene-based detection method, which successfully identifies F-like transfer operons regardless of contig fragmentation.

In the following text, we use the F-like transfer operon both as a marker to detect the presence of the F-like plasmid and to assess transfer potential.

We first set out to estimate the prevalence of F-like plasmids in our *E. coli* genomes. Using a minimum threshold of five F-like transfer genes to mark an F-like plasmid as present, we found a prevalence of 65.5% in the entire dataset. We found significant differences in F-like plasmid prevalence between the three animal environments ($\chi ^{2}(2) = 152.00, P < 2e-16$; Fig. 2A) with bovine at 44.75%. swine at 65.5%, and poultry at 86.25%. These prevalence estimates were robust to a wide range of marker gene thresholds (Fig. 2B). At the low end (<4) there is a sub-population of bovine *E. coli* with three transfer genes that inflate the number of F-like transfer operons identified. On the high end, prevalence begins to drop quickly above 27 marker genes in all three animal environments. Interestingly, this number is larger than the 23 genes with a demonstrated function in F conjugation [3, 7, 8]. Only 2.67% of all genomes contained all 35 marker genes found in the classical F plasmid.

**Figure 2:**
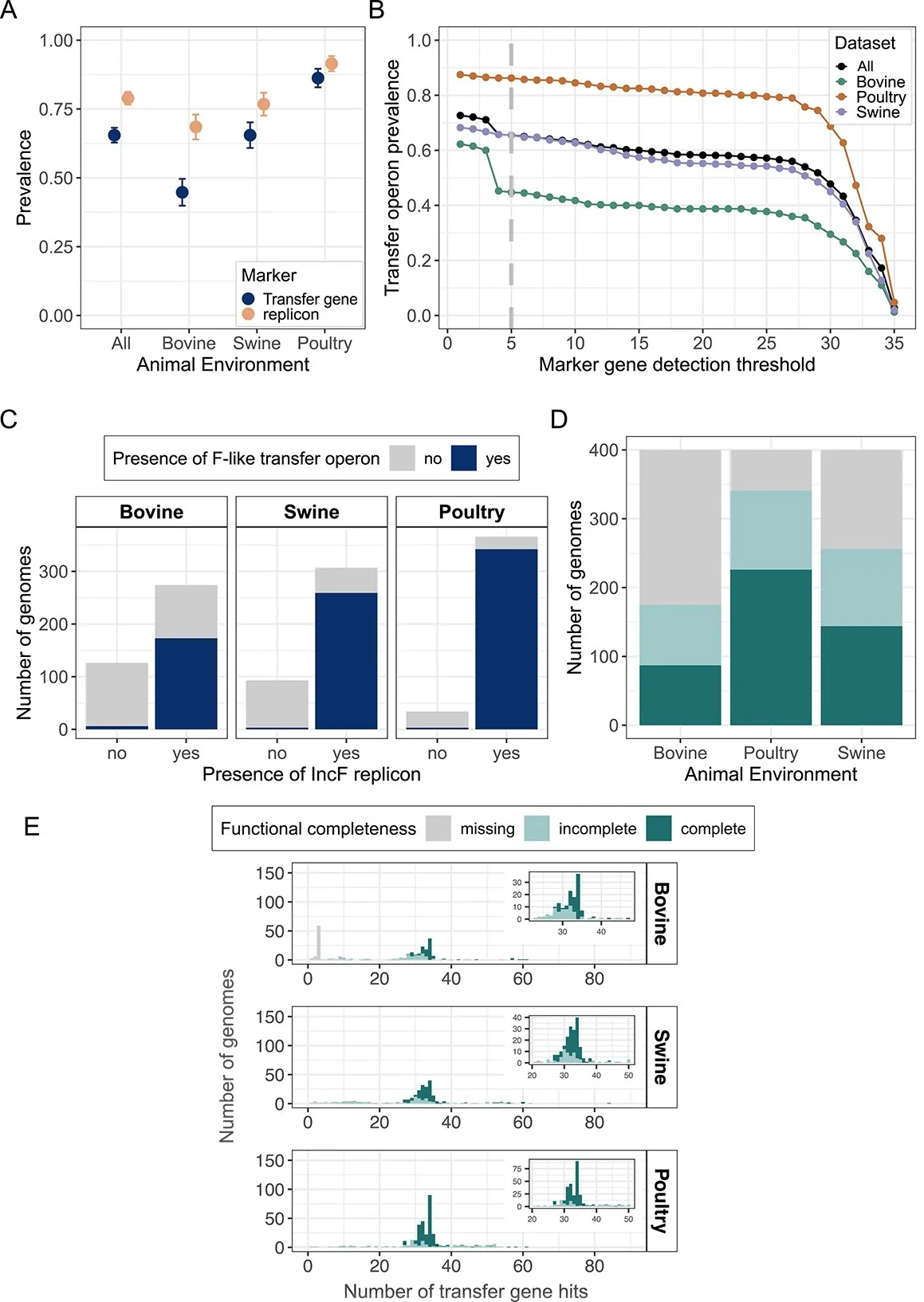
**A.** Prevalence of F-like plasmids estimated using transfer genes or replicons as markers across the entire dataset (all; *n = 1200*  *E. coli* isolates) and within each animal environment (*n = 400* each). A minimum of five different transfer genes were necessary to call a plasmid ‘present’ in a genome. **B.** Estimates of F-like transfer operon prevalence across our entire dataset and in each animal environment as a function of the number of genes required to mark an operon as present. The vertical dashed line indicates the five gene detection threshold used in further analyses. **C.** Proportion of *E. coli* genomes containing F-like transfer operons or not , given the presence or absence of an IncF replicon in a genome. **D.** Proportion of *E. coli* isolates from each animal environment that have F-like transfer operons that are ‘complete’ (all 23 essential F-like transfer genes), ‘incomplete’ (between 5 and 22 essential genes), or ‘missing’ (less than five essential genes). **E.** Number of F-like transfer genes detected per genome across the three animal environments. Genomes are coloured by the functional completeness category they were assigned. The insets show a zoom-in of the distribution between 20 and 50 transfer gene hits. Colour legends for panels D and E are the same.

To compare with past estimates of plasmid prevalence in environmental datasets, we also estimated the prevalence of IncF replicons in our dataset (2A). Replicons are loci involved in replication (Fig. 1A), and are very commonly used to classify plasmids into families that reflect incompatibility groupings. Using the replicon typing tool PlasmidFinder, IncF replicon prevalence was found to be 78.92% in the entire dataset and 68.5%, 76.75%, and 91.50% in bovine, swine, and poultry, respectively. This difference was statistically significant between animal environments: $\chi ^{2}(2) = 65, P < 7e-15$. The replicon-based prevalence estimates were always higher than those based on the F-like transfer operon (Fig. 2A). Absence of a replicon was associated with absence of the transfer operon (Fig. 2C)). The inverse, however, was not necessarily true (Fig. 2C) as 36.86%, 15.64%, and 6.56% of bovine, swine, and poultry *E. coli* that contain IncF replicons were lacking F-like transfer operons.

Our estimate of replicon-based prevalence of 78.92% is similar (though slightly lower) to previously reported prevalence estimates in livestock and human commensals (85.2% and 82.19%, respectively [31, 32]; Table 1), and higher than estimates reported for wastewater treatment plants and human bloodstream infections (73.5% and 73.51%, respectively [31, 33]). However, our transfer-gene-based prevalence estimate (65.5%) is lower than replicon-based prevalence estimates reported across all datasets. Interestingly, both our transfer-gene- and replicon-based prevalence estimates vary across the three animal environments, in contrast to a livestock dataset from England [31], which reported similar replicon-based prevalences across the same livestock environments (Table 1).

**Table 1:** Reported F-like plasmid prevalence estimates for *E. coli* isolated from different environments. In this study, prevalence was estimated using two different types of marker genes: IncF replicons or transfer genes (here with a detection threshold of at least five transfer genes). 95% confidence intervals, estimated using a normal approximation to the binomial distribution, were reported for the prevalence estimates in this study.

Study	Niche	Sub-Niche	Sample size (*n*)	F-like plasmid prevalence	Gene marker	Tool
Shaw *et al*. [31]	Wastewater		83	0.73	Replicon	MOB-typer (replicon typing)
	Livestock		378	0.85		
		Cattle	140	0.85		
		Pig	102	0.85		
		Sheep	136	0.85		
Stephens *et al*. [32]	Human commensal *E. coli* from healthy adults		101	0.82	Replicon	PlasmidFinder (replicon typing)
Reid *et al*. [33]	Clinical (bloodstream infections)		4711	0.74	Replicon	pMLST (replicon typing)
**This study**	**Livestock**		**1200**	**0.79 [0.77, 0.81]**	**Replicon**	PlasmidFinder (replicon typing)
		**Bovine**	**400**	**0.69 [0.64, 0.73]**		
		**Poultry**	**400**	**0.92 [0.89, 0.94]**		
		**Swine**	**400**	**0.77 [0.73, 0.81]**		
			**1200**	**0.66 [0.63, 0.68]**	**Transfer gene**	F-like transfer gene detection using tblastn and Bakta
		**Bovine**	**400**	**0.45 [0.40, 0.50]**		
		**Poultry**	**400**	**0.86 [0.83, 0.90]**		
		**Swine**	**400**	**0.66 [0.61, 0.70]**		

### Animal environments differ in F-like transfer operon completeness

The inconsistency between the replicon- and transfer-gene-based prevalence estimates suggests that the three animal environments differ in the types of F-like plasmids present, specifically in the completeness of their transfer operons. Completeness of the transfer operon is a proxy for functionality, as plasmids lacking essential transfer genes cannot conjugate on their own [8, 24]. We classified each genome into three completeness categories, based on the presence of 23 F-like transfer gene markers with an identified function in conjugation (Figs 1A, Table S1). Mutations in these genes have been shown to block or reduce transfer [3, 7, 8]. We, henceforth, refer to this set of 23 genes as ‘essential genes’. Genomes were classified as ‘missing’ F-like transfer operons if less than five essential genes were detected, ‘incomplete’ for *≥*5 and <23 essential genes, and ‘complete’ if all essential genes were detected. We note that this classification is not influenced by genome quality: we find no meaningful variation in genome completeness or contamination scores across these groups (Fig S2B,C).

We find a statistically significant association between animal environment and functional completeness ($\chi ^{2}(4)= 165, P < 2 \times 10^{-16}$, Cramér’s V effect size = 0.26 [0.22, 0.30]). Poultry isolates contained significantly more complete F-like transfer operons (Supplementary Table S2), while bovine isolates were more often missing F-like transfer operons than expected based on independence of variables (Fig. 2D). Interestingly, the proportion of functionally incomplete F-like transfer operons is similar among the three animal environments.

We compared our classification results with the mobility typing scheme used in Coluzzi *et al.* [22], which relies on the relaxase and a small number of transfer genes to distinguish conjugative, mobilizable, and non-mobilizable plasmids (Supplementary Text). In this case, we found the same rank order of prevalence of conjugative plasmids across the three animal environments (Poultry > Swine > Bovine; Fig. S5). However, the absolute prevalences increased because the scheme of Coluzzi *et al.* requires fewer transfer genes to classify a transfer operon as conjugative.

We then assessed whether operon completeness is associated with specific plasmid identities. To do so, we used the MOB-recon tool from the MOB-Suite package to assign plasmid cluster identities to the F-like contigs in each draft genome (hence termed F-like plasmid clusters, Supplementary Text). Only 38.5% of genomes containing an F-like transfer operon (either complete or incomplete, *n=786*) could be assigned a plasmid cluster. Notably, we find that the same F-like plasmid cluster can be mapped to both functionally complete and incomplete transfer operons (Fig. S6), implying that the same plasmid backbone can differ in their putative ability to conjugate.

To better understand how operon completeness relates to the the total number of transfer genes hits found in each genome, we counted all occurrences of the 35 transfer gene markers in each isolate and compared the distribution of transfer genes across the three environments. Here, multiple occurrences of a transfer gene were counted separately. We find a similar distribution of transfer genes in each animal environment (Fig. 2E). The distribution is bimodal, with most isolates either completely lacking an F-like transfer operon or having between 23 and 35 F-like transfer gene hits. The exception is a peak in Bovine *E. coli* at three F-like transfer genes (*n = 59* isolates). Interestingly, 34.1% of the isolates with *≥*23 genes—the size of our essential gene set—have functionally incomplete transfer operons. We also observe a long tail of isolates containing >35 transfer genes (9.17% of all isolates), likely indicative of the presence of multiple F-like transfer operons.

We also checked how transfer gene hits were distributed across contigs in a genome and whether there are associations to operon completeness (Fig. S4A). Over 66% of genomes with complete F-like transfer operons have transfer genes detected on just one contig. Complete genomes had statistically fewer contigs with transfer genes compared with incomplete genomes (Wilcoxon rank-sum test with continuity correction, *W=47 564*, $P<2 \times 10^{-16}$). We observe strong conservation of gene synteny even when genes are split across multiple contigs, with 92% of genomes (in which gene synteny can be assessed, *n=872*) containing contigs with transfer genes ordered as found on the F plasmid (Fig. S4B).

### Differences in plasmid prevalence across animal environments persist after correction for confounders

We next asked whether the difference in prevalence of complete F-like transfer operons across the three animal environments might be confounded by significant differences in *E. coli* phylogenetic background (PhG A-F), sample source (whether the sample came from food or the live animal), presence of antimicrobial resistance genes (ARG presence yes/no), isolate, or sampling year (before 2017; 2017 and later) (Figs 3A, S7 and Table S3). We used logistic regression to model the odds of harbouring complete F-like transfer operons with these covariates and their interaction terms (Fig. 3B). The model with the lowest AIC included animal environment, PhG, sample source, and ARG gene presence as main effects (Table S4) and an interaction between PhG and ARG presence (Table S5). Sampling year is not included as a covariate, which is additionally supported by the observation that we find no difference in F-like transfer operon completeness in E. coli sampled before 2017 and after 2017 ($\chi ^{2}(1) = 3.63, P =.057$, Cramér's V effect size = 0.05 [0.00, 0.11]), Fig. S7).

**Figure 3:**
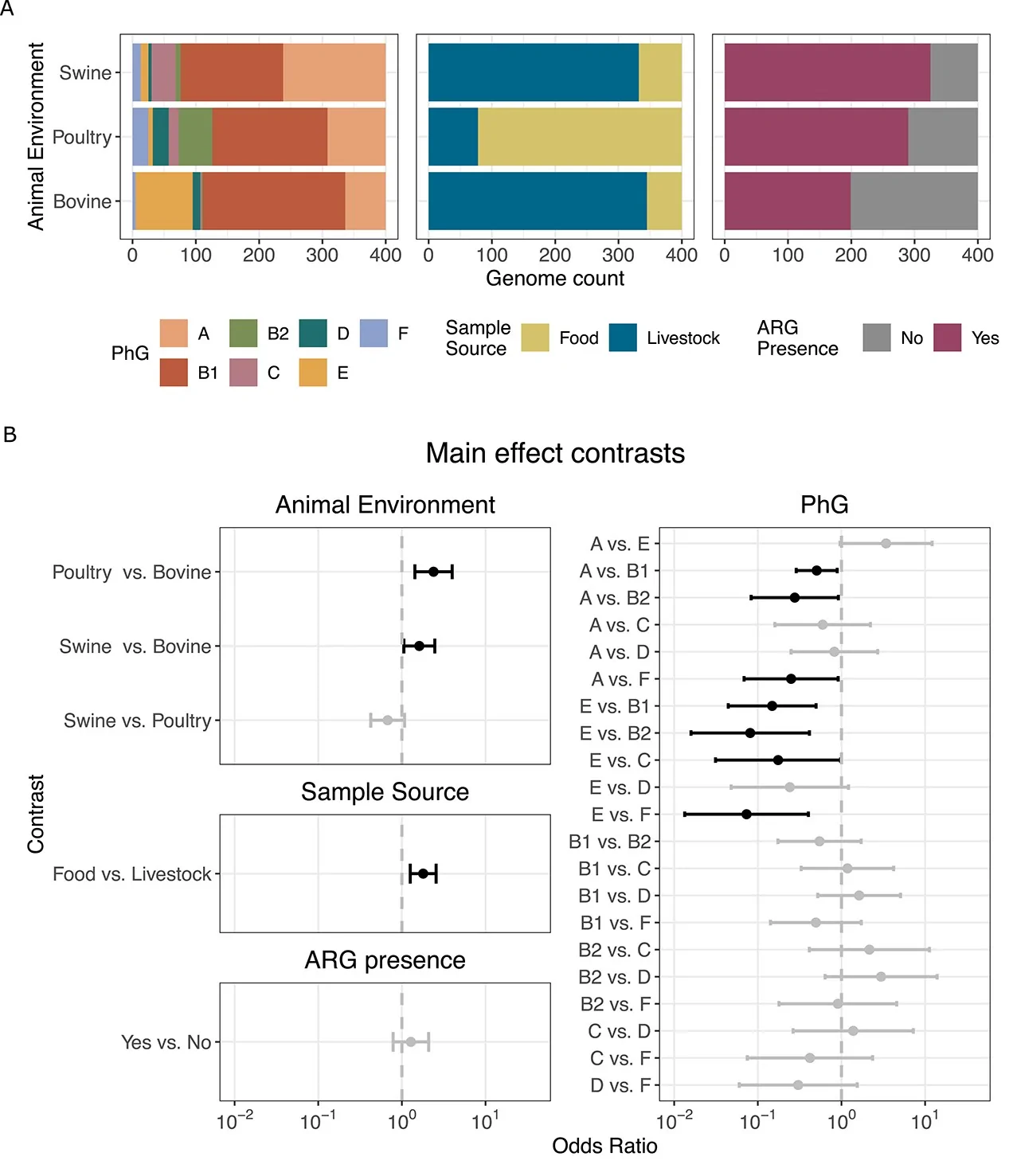
**A.** Left: *E. coli* phylogroup (PhG) abundances per animal environment. Middle: proportion of genomes isolated from a given sample source and animal environment. Livestock refers to the live animal, while Food refers to any sample recovered from food products. Right: presence of at least one ARG in isolates from a given animal environment. **B.** Odds ratio of harbouring a complete F-like plasmid in one group vs. the other after adjusting for all other covariates. The x-axis is on a log scale, and the error bars indicate 95% confidence limits. Contrasts that were significant (*P*<.05) after correction for multiple testing are depicted in black.

Even after adjusting for these confounders, we find significant differences in the odds of harbouring a complete F-like plasmid across the different animal environments. Both poultry and swine *E. coli* have higher odds than bovine isolates (OR: 2.39 [1.43, 4] and 1.62 [1.06, 2.47], respectively). Swine *E. coli* has lower odds compared with poultry, although the difference is not statistically significant (OR: 0.68 [0.42, 1.08]).

There are also independent effects of the sample source and phylogroup on the odds of harbouring a complete F-like transfer operon. Food isolates have significantly higher odds of harbouring a complete F-like operon compared with livestock isolates (OR = 1.8, 95% CI: 1.26, 2.57), a difference that is not specific to a particular animal environment. Among the different phylogroups, all significant differences involved either PhG A or E (Fig. 3B). We find significantly lower odds of a complete F-like operon in PhG A than in B1, B2, or F (0.51 [0.29, 0.89]; 0.28 [0.09, 0.92]; 0.25 [0.07, 0.91], respectively) and significantly lower odds in PhG E than B1, B2, C, or F (0.15 [0.04, 0.5]; 0.08 [0.02, 0.41]; 0.17 [0.03, 0.98]; 0.07 [0.01, 0.4], respectively).

Interestingly, across all genomes we find no significant difference in complete F-like operons between *E. coli* that carry resistance genes and those that do not (Fig. 3B). Yet this picture changes when we take into account the interaction between resistance carriage and phylogroup (Supplementary Figure S8). Within PhG A, the odds of harbouring a complete F-like operon were significantly higher in isolates carrying resistance genes (2.79 [1.54, 5.04]). In PhG B2 instead, the odds were significantly lower in isolates carrying resistance genes (0.17 [0.04, 0.69]).

A sensitivity analysis repeating the logistic regression procedure on a dereplicated dataset (1160 genomes; excluding isolates with *≥*99.99% ANI, identical sampling metadata, and similar plasmid content; Fig. S3C, S9) confirmed that most observed effects remained robust. However, we note that the difference between swine and bovine isolates falls below statistical significance (Fig. S9A).

Therefore, we show that animal environment differences in F-like operon presence remain significant even after accounting for confounding by phylogroup, presence of resistance, and sample source.

### Transfer gene frequencies are correlated with gene function

We asked whether differences in F-like transfer operon prevalence between animal environments extend to more fine-grained differences in transfer gene presence/absence between animal environments. We estimated individual gene frequencies for each of the 35 transfer genes relative to the total number of genomes with at least one transfer gene in a given environment (Fig. 4). This is to avoid inflating gene frequency differences due to the different baseline frequency of F-like plasmid presence in different environments. Even with this definition, the median gene frequencies differ by animal environment (62% in bovine, 81% in swine, and 91% in poultry, respectively), reflecting differences in functional completeness across the environments. To focus on the prevalence of specific genes relative to the rest of the operon, we interpret patterns of transfer gene content relative to the median gene frequency of the transfer operon in that animal environment. Frequencies at or higher than the environment-specific median gene frequencies suggest that the gene is important in the conjugation process, whereas frequencies lower than the environment-specific median suggest non-essentiality.

**Figure 4:**
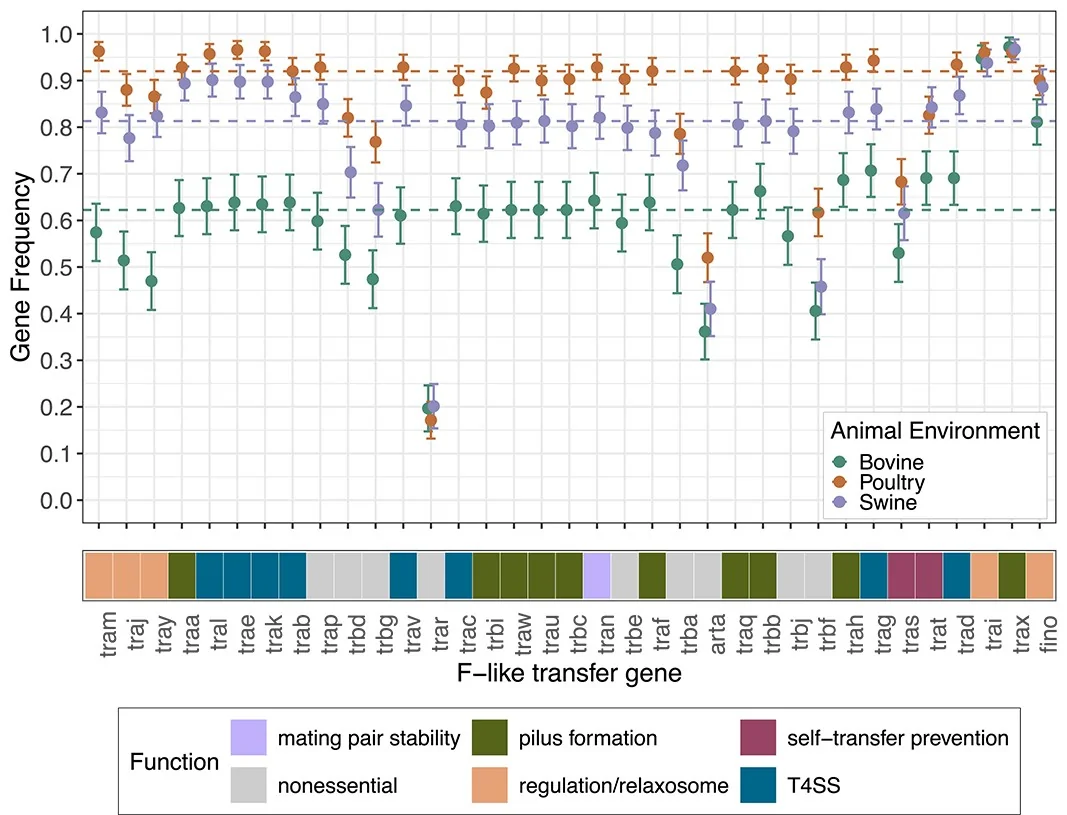
Gene frequencies of individual transfer genes in *E. coli* genomes isolated from bovine , poultry , and swine . Transfer genes are coloured by their role in conjugation. Gene frequencies were calculated relative to the total number of genomes from each animal environment (*n=*400) that contained at least one transfer gene hit. The error bars indicate 95% confidence intervals, computed using a normal approximation to the binomial distribution. The coloured dashed lines refer to median gene prevalences within each animal environment.

We find that the relative transfer gene frequencies are mostly independent of the animal environment, and are primarily determined by their functional role in conjugation. Genes previously described as essential for conjugation have higher frequencies than those that are non-essential (Fig. 4). The relaxase *traI* is present at frequencies over 90% in all three animal environments, indicative of its important role in conjugation and mobilization. The other pilus formation genes and genes involved in the T4SS have gene frequencies close to or higher than the median gene frequency in each animal environment (Fig. S10). Instead, non-essential genes typically have frequencies lower than the median gene frequencies, indicating that these genes are often missing in operons. Two exceptions are *traP* and *trbE*, non-essential genes with frequencies closer to the median gene frequencies in each niche, hinting that they may play a functional role in natural environments. Transfer gene *traR*, described to be non-essential, has the lowest gene frequencies in all three animal environments, at around 19%.

Interestingly, regulatory genes like *traM, traJ, traY*, and *finO* and self-transfer prevention genes *traS* and *traT* have variable patterns of presence in the three animal environments (Fig. 4, Supplementary Figure S10), potentially indicating environment-specific diversity patterns.

### Poultry *E. coli* host a larger diversity of conjugative plasmids

Since *E. coli* often carry multiple plasmid types simultaneously [54], we asked whether we also find differences in the prevalence of non-F conjugative systems between the animal environments. To do so, we identified which MPF types are present in each isolate using geNomad [48]. Because MPF types are defined based on sequence homology of components of the T4SS and other proteins involved in transfer, they directly reflect the types of conjugative plasmids present in the isolate. We find that more than a third of the isolates (35.91%) carry more than one MPF type (Fig. S11).

We found significant differences between the animal environments in the number of non-F MPF types carried per isolate ($\chi ^{2}(2) = 66.22$, *P <.001*, Kruskal–Wallis rank-sum test). In terms of carriage, poultry *E. coli* harbour significantly more non-F MPF types than bovine and swine *E. coli* (Figs S12, 5A). Swine and bovine do not differ significantly in their number of non-F MPF types. Interestingly, in both poultry and swine, we find no difference in non-F MPF type carriage between isolates that carry complete, incomplete, or lack F-like operons ($\chi ^{2}(2)=2.72$, *P=.26*, $\chi ^{2}(2)=1.02$, *P=.60,* respectively, in poultry and swine). Interestingly, bovine isolates missing F-like operons seem to carry fewer plasmids of other types too (missing vs. incomplete *P<.01*; missing vs. complete *P<.05*). Generally, however, the presence of F-like plasmids does not seem to influence the number of other conjugative plasmids carried by these *E. coli* isolates.

**Figure 5:**
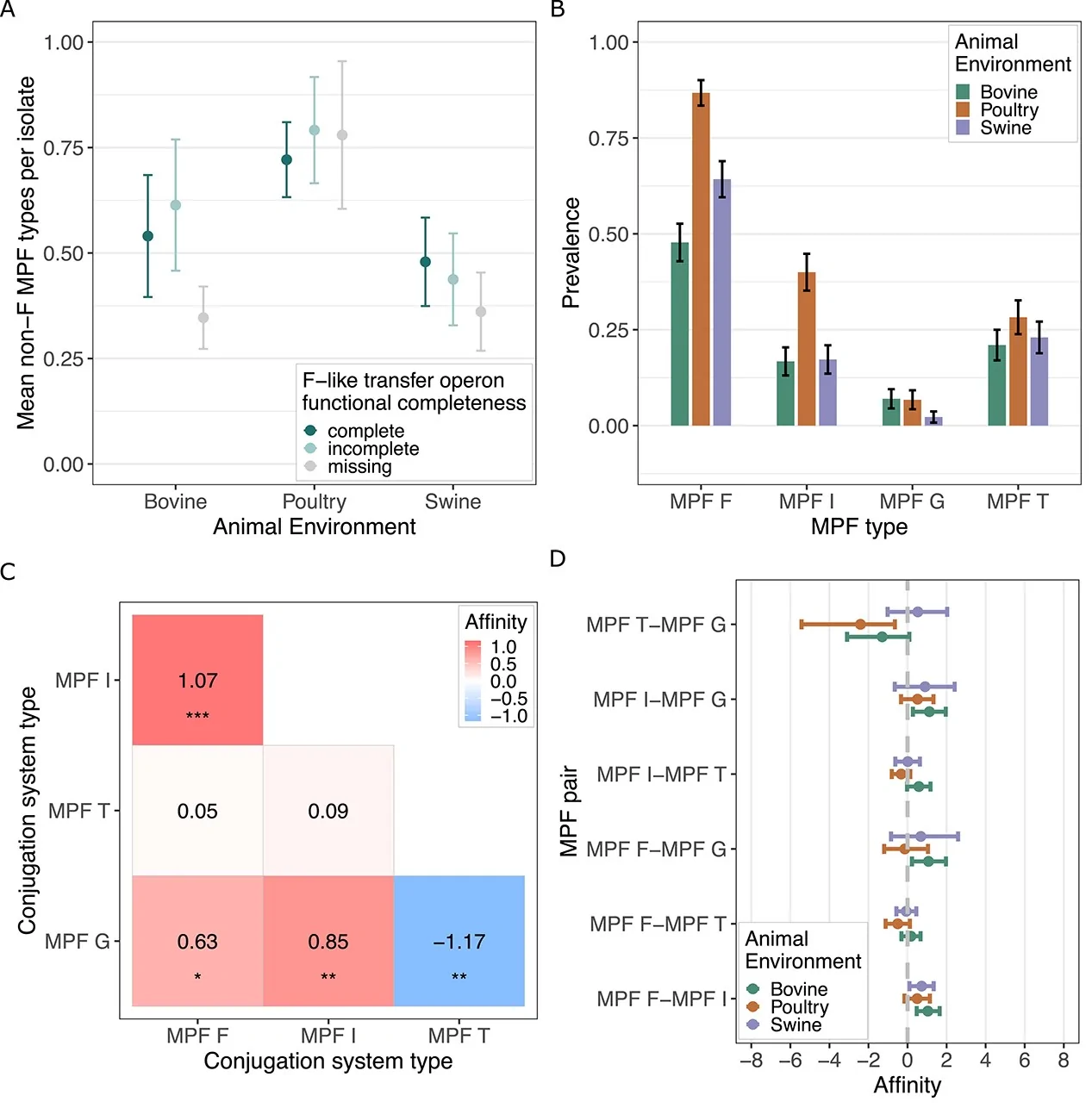
**A.** Mean number of non-F MPF types per isolate across animal environments and F-like completeness categories. Error bars are 95% confidence intervals around the mean. **B.** Prevalences of MPF types across animal environments. MPF prevalence is computed as the proportion of genomes where at least three distinct genes specific to an MPF type were detected. The error bars indicate 95% confidence intervals, computed using a normal approximation to the binomial distribution. **C.** Affinity matrix describing the odds of finding pairs of conjugation system types together in a isolate. The positive values indicate higher odds of finding a pair together than the null expectation of random assortment. The negative values indicate higher odds of finding a pair in two different isolates than the null expectation. A 0 value indicates co-occurrence as expected based on individual frequencies (null expectation). Significance levels: ^*^P*≤*.05, ^*^^*^P*≤*.01, ^*^^*^^*^P*≤*.001. **D.** Affinity of MPF pairs across animal environments.

We looked at the prevalences of the individual MPF types across the animal environments (Fig. 5B). In addition to the significant differences in prevalences of MPF F across animal environments, we also find significant differences in the prevalence of MPF I ($\chi ^{2}(2)=75.94$, *P<.001*) and MPF G ($\chi ^{2}(2)=11.323$, *P<.01*). Poultry isolates have a higher prevalence of MPF I than swine and bovine isolates. This higher prevalence of MPF I was the driver of overall higher numbers of non-F MPF types in poultry (Figs 5A, S12).

### Co-occurrence patterns of conjugative systems are similar in all three environments

We wanted to test whether specific conjugative plasmid types co-occur with F-like conjugative plasmids in an isolate and whether these patterns differ by animal environment. We assessed whether pairs of MPF types are detected together in an isolate more or less frequently than expected based on the prevalence of individual MPF types and a null distribution of independent random assortment [55](Supplememtary Text). As a test of our method, we first assess the co-occurrence between MPF and relaxase types in the entire dataset. Since a functional conjugation system requires both an MPF system and a relaxase, we expect to find mostly positive associations between MPF and MOB types. Indeed, we find 12/20 MPF-MOB pairs that are significantly positively associated (Fig. S13).

We next assessed co-occurrence between pairs of MPF types in the entire dataset (Fig. 5C), focusing here on the associations with MPF F. Plasmid types MPF I and MPF G co-occur more often with MPF F than expected by chance. On the other hand, MPF T is not positively or negatively associated with MPF F. These patterns also persist at the level of the individual animal environments (Fig. 5D). This suggests that regardless of the environment, similar conjugative plasmids tend to be found together with F-like plasmids. In fact, patterns of co-occurrence are similar across the animal environments for almost all pairs of MPF types assessed.

## Discussion

In this work, we investigated the genetic diversity of F-like transfer operons in *E. coli* from livestock environments, to understand the distribution of transfer potential in natural bacterial populations. We used F-like transfer gene presence to measure transfer operon completeness as a proxy for transfer potential. This novel approach showed that livestock environments differ in their prevalence of complete F-like transfer operons, with a significantly higher frequency found in poultry (56%), than swine (36%) or bovine (22%) environments (Fig. 2D). Notably, this difference in transfer potential persists even after accounting for possible confounders such as *E. coli* phylogenetic background, presence of antibiotic resistance genes, and isolation from food or the live animal (Fig. 3B, S8).

While many studies use the replicon to estimate plasmid presence in natural environments (e.g. [30–33]), we find that replicon-based plasmid prevalences are systematically higher than our transfer-operon-based prevalence estimates. This suggests that studies that rely only on replicons to assess plasmid prevalence may overestimate the transfer potential of F-like plasmids in natural environments. Indeed in our study, 19.6% of the isolates containing an F-like replicon were missing F-like transfer operons entirely and 32.42% were lacking essential genes required for self-conjugation (Fig. 2C).

Our estimates of functional F-like plasmid prevalence provide insight into the distribution of plasmid mobility at the population level and how this varies by environment. Transitions in plasmid mobility are thought to be frequent, mostly involving deletions or pseudogenizations of transfer genes, leading to a loss of the ability to self-conjugate [22, 23]. While we observe differences in the frequencies of genomes with complete or entirely missing F-like transfer operons, the frequency of incomplete F-like operons is similar across the three environments (*∼*25%; Fig. 2D). This suggests that the per-plasmid rate of transitioning from complete to incomplete or incomplete to missing differs across the three host environments. To understand how different environments can maintain different frequency distributions of the three mobility classes, it will be important to model selection on mobility and the rate of transitions between classes.

This study also sheds light on the relative roles of environment and phylogenetic background in shaping plasmid diversity. In our collection of livestock-associated *E. coli*, we found that the variance in F-like transfer potential is better explained by the animal environment than the *E. coli* phylogenetic background (Fig. 3B). This is consistent with *E. coli* sampled from diverse environments in Australia [51], where MGE-associated gene variation was explained more by environment than *E. coli* phylogroup. However, it contrasts with what has been observed at broader taxonomic scales. In a study on plasmids from diverse bacterial isolates across multiple environments, bacterial host phylum explained more variation in plasmid relaxase genes than the isolation environment [35]. The distribution of plasmid mobility has also been found to vary across bacterial taxa, independent of the environment they are found in [22]. These observations suggest that at broader phylogenetic scales, lineage-specific constraints most strongly dictate plasmid diversity, whereas at the level of a single bacterial species environment-related effects can become more apparent. Further comparative studies within bacterial species other than *E. coli* will be necessary to determine how general this scale-dependent pattern is.

We find that poultry isolates are distinct from both swine and bovine isolates, hosting more conjugative plasmids per isolate (Figs 5A,B, S12). Our observation is consistent with studies that reported enrichment of MGEs in poultry isolates [51, 56]. In addition to this enrichment in MGEs, poultry isolates have also been found to be enriched in virulence and resistance genes [51]. This stand-alone position of poultry could be due to the specific selection-pressures found in poultry farming, where heavy antibiotic use is often the norm [56], or inherent differences in animal physiology [57] and microbiome [58, 59]. Distinguishing between these different hypotheses would require targeted sampling of poultry farms coupled with farm-specific metadata on antibiotic consumption.

Despite differences in the frequencies of conjugative plasmids between the environments, the patterns of transfer gene presence and absence are similar (Figs 4,S10) and correlates with functional importance in conjugation. This suggests that selection for a given transfer gene is similar across the environments. We find exceptions to this pattern in essential transfer genes involved in regulation and exclusion, which display the considerable variability in gene frequencies across environments, suggesting different environments might have different preferred regulatory variants and exclusion potential. A previous study looking at F-like plasmid transfer operons in *Enterobacteriaceae* across a diversity of environments found alternate regulatory configurations in non-*E. coli* species [24]. That we find such patterns also in a single bacterial species suggests that the regulation of conjugation is also modulated by environment-specific selection pressures, and not solely constrained by phylogenetic background.

Our gene-based detection protocol assumes that the presence of F-like transfer genes in the genome is a sufficient condition for the formation of a conjugation apparatus, even if the genes forming the operon are on different contigs. This assumption is supported by genetic complementation studies, which demonstrate that a plasmid’s non-functional transfer gene can be rescued by introducing a functional variant on a second plasmid [60–62]. Thus, a functional conjugation system can still assemble as long as the necessary genes are present and functional, even if the genetic components are on different plasmids. A recent study has also shown that co-resident pED208 and F plasmids (both F-like plasmids) missing essential transfer genes can exchange subunits to build a functional chimeric conjugation system [63]. Our approach demonstrates the viability of draft genomes, which make up the vast majority of public genome databases, as a resource for environmental plasmid surveillance.

Together, our results demonstrate that the environment is a major driver of variation in F-like plasmid prevalence and mobility potential across *E. coli* populations. While our analyses focused on F-like plasmids, they are easily transferable to other MGEs. Such careful studies of plasmid mobility at the level of individual transfer genes will be essential to arrive at environment-specific assessments of transfer potential, allowing us to predict how plasmids spread in natural bacterial populations.

## Supplementary Material

F_div_revised_supp_ycag256

## Data Availability

Code and data files (including the accession numbers of genomes) used to reproduce the analyses and figures can be found in this github repository https://github.com/Sneha-Sundar/F_div_paper.
