# Supplementary material for: Transfer potential of F-like plasmids in Escherichia coli differs by animal environment
Source: ISME Commun. 2026 Sep 6;6(1):ycag256. doi: 10.1093/ismeco/ycag256 (PMC13639103; doi:10.1093/ismeco/ycag256)
Supplement: Supplementary file 1 [file f_div_revised_supp_ycag256.pdf]

S1 Supplementary Figures

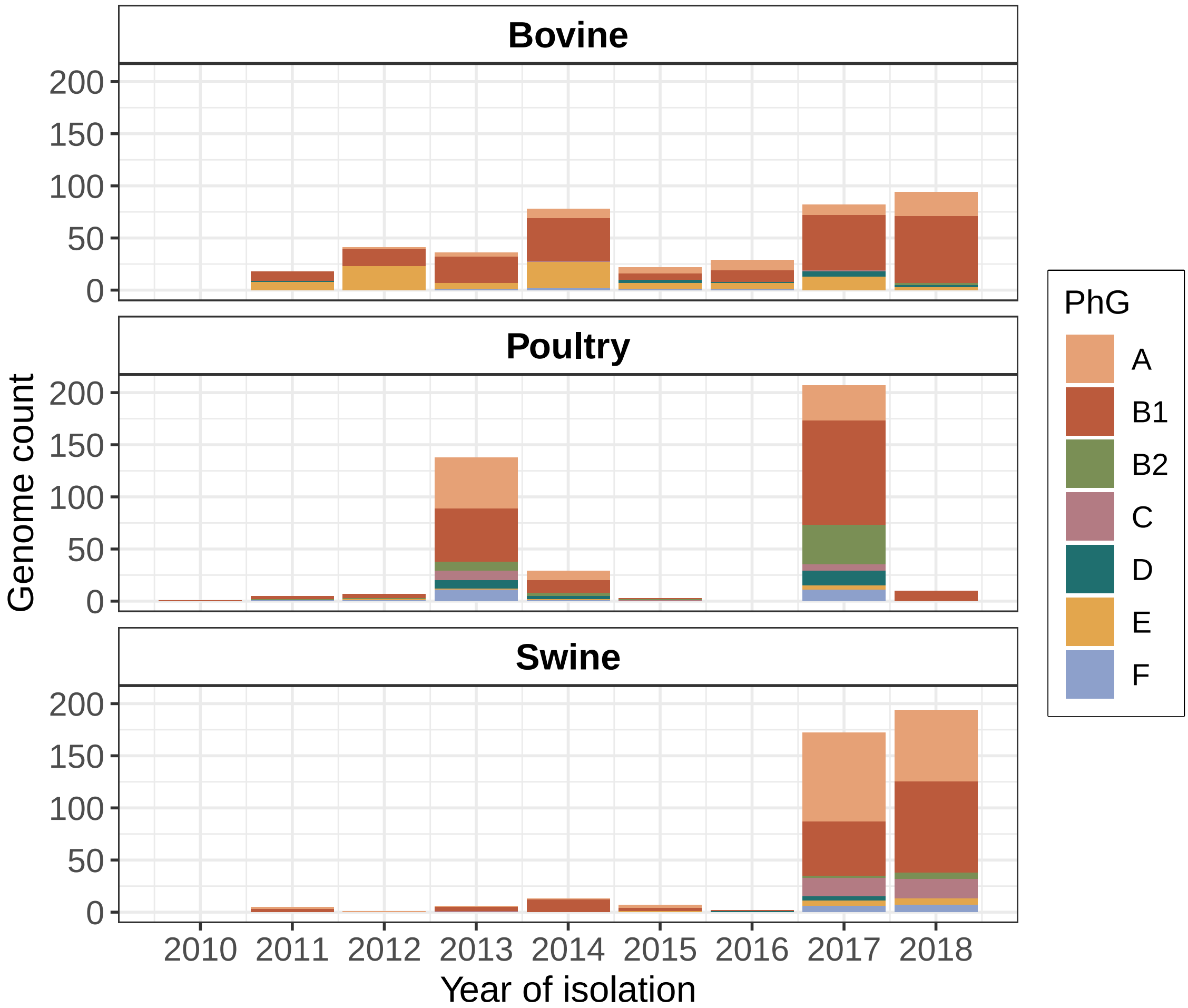

Figure S1: The distribution of our 1200 *E. coli* genomes across animal environments (row; n=400 per animal), isolation date (x-axis), and phylogroup (color).

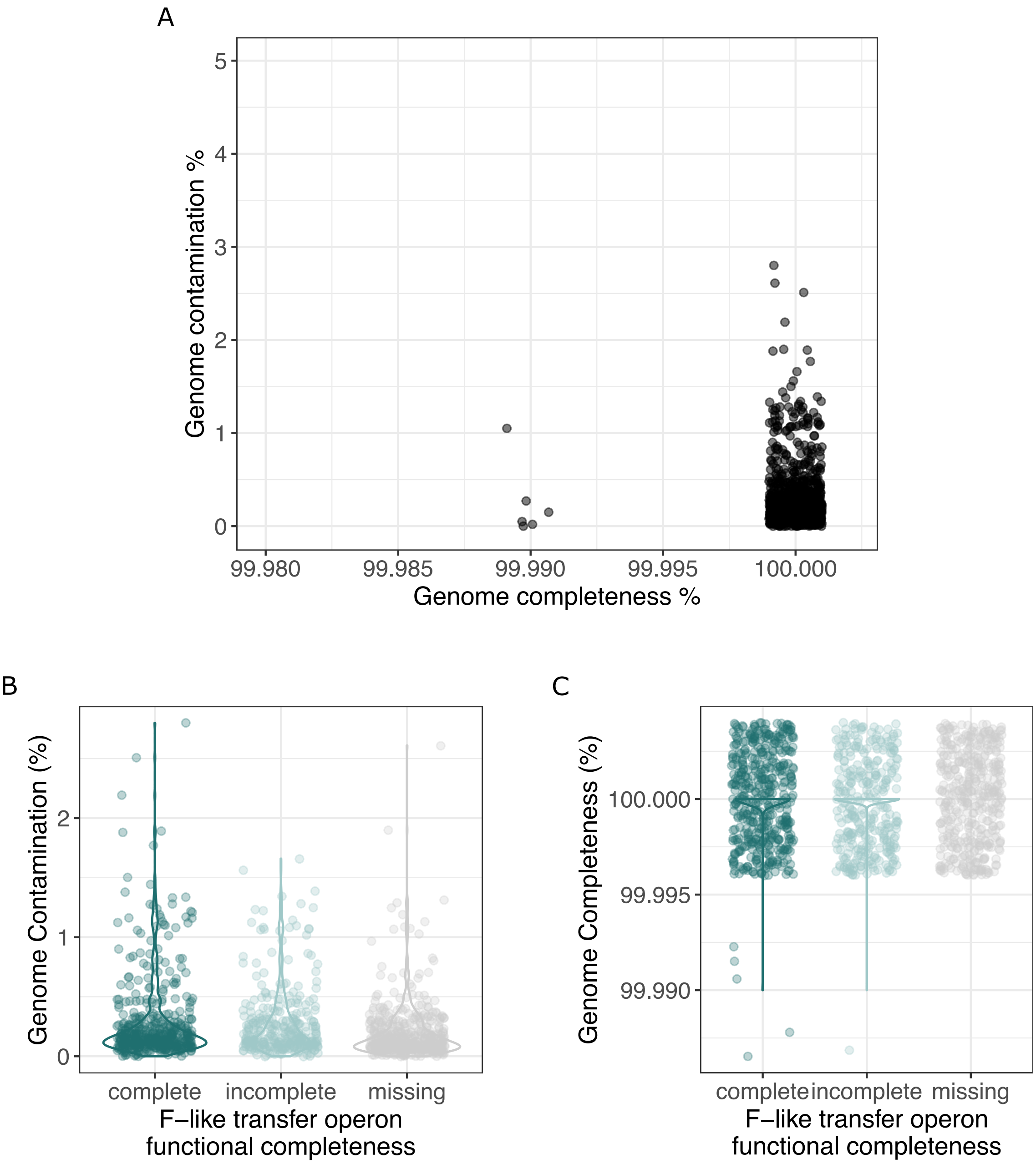

Figure S2: **A.** Genome completeness and contamination scores of all genomes assessed using CheckM2 [1]. All genomes had ≥99.9% completeness and <3% contamination and meet the standard criteria for high quality draft genomes (≥95% completeness, ≤5% contamination [2]) **B.** Contamination scores for genomes with complete, incomplete and missing F-like transfer operons **C.** Completeness scores for genomes with complete, incomplete and missing F-like transfer operons

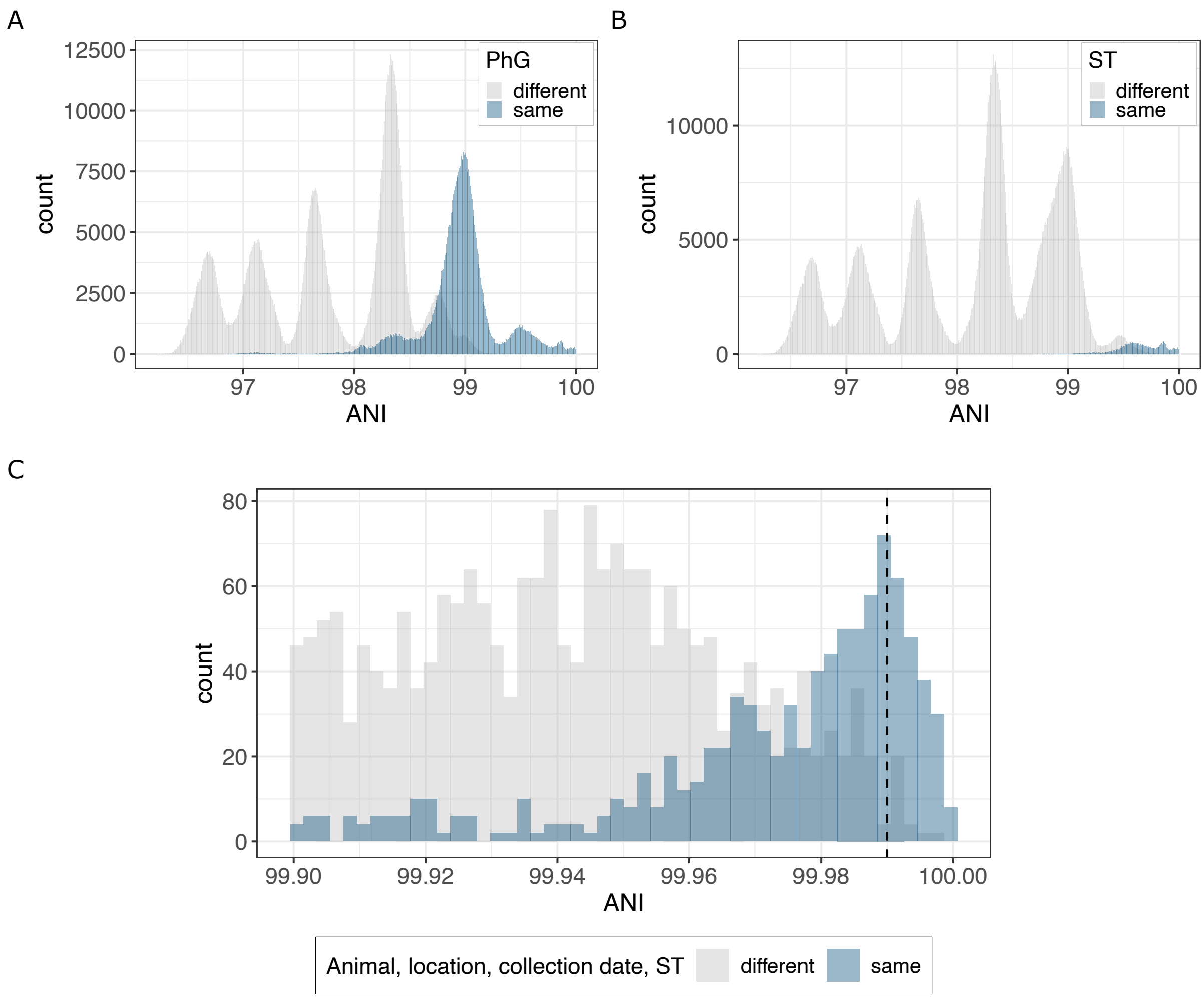

Figure S3: Pairwise Average Nucleotide Identity (ANI) distributions. **A.** Intra-phylogroup (blue) vs. inter-phylogroup (grey) pairs. **B.** Intra-sequence type (blue) vs. inter-sequence type (grey) pairs. **C.** Highly similar pairs (ANI $\geq$ 99.9%) sharing identical animal environments, locations, collection dates, and sequence types. The dashed line represents the recommended threshold (99.99%) for strain-level classification [3, 4].

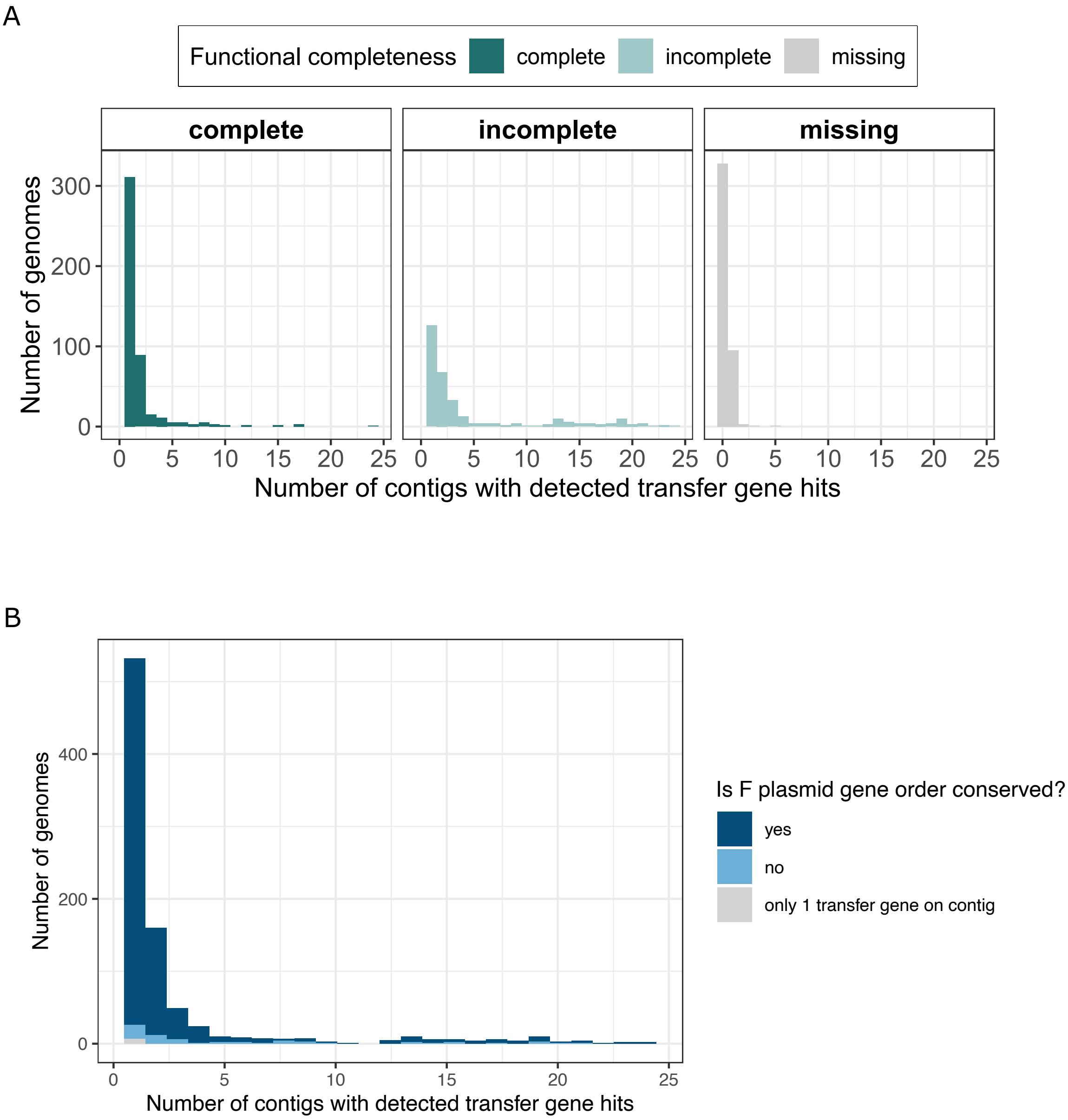

Figure S4: **A.** Distribution of the number of contigs in which transfer genes are detected in genomes with at least 1 detected transfer gene (n = 872). Colour indicates the proportion of genomes in which F plasmid gene order is conserved in all contigs with at least 2 transfer gene hits. **B.** Distribution of the number of contigs with detected transfer genes within each functional completeness category (all 1200 genomes assessed).

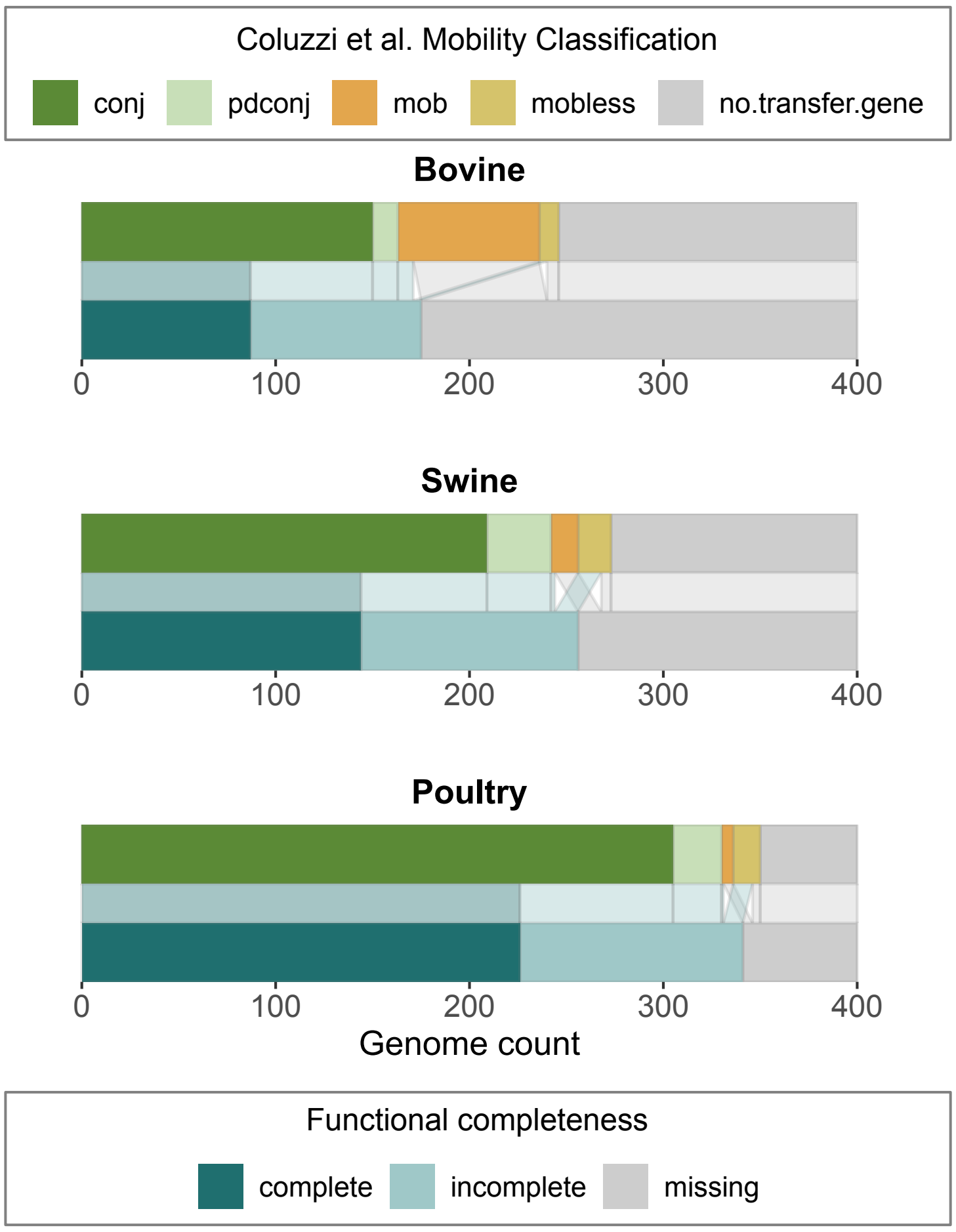

Figure S5: Proportion of *E. coli* isolates with F-like transfer operons within each animal environment, as estimated using either the mobility typing scheme of Coluzzi et al. [5] (top row in each panel) or the functional completeness metric defined in this study (bottom row). Mobility typing in Coluzzi et al. uses the presence of the relaxase and a minimum number of other conjugation-related genes to classify a plasmid: conjugative (conj: contains relaxase, VirB4, T4CP and at least 3 MPF proteins), mobilizable (mob: contains relaxase but missing the required number of other components to be conjugative), mobless (lacking the relaxase) or partially decayed conjugative (pdCONJ: a subcategory of MOB plasmids with more than 5 conjugation proteins excluding the relaxase) or as having no F-like transfer gene (no.transfer.gene). The functional completeness categories used in this study were based on a set of 23 essential transfer genes: 'complete' (all 23 essential F-like transfer genes), 'incomplete' (between 5 and 22 essential genes) or 'missing' (less than 5 essential genes).

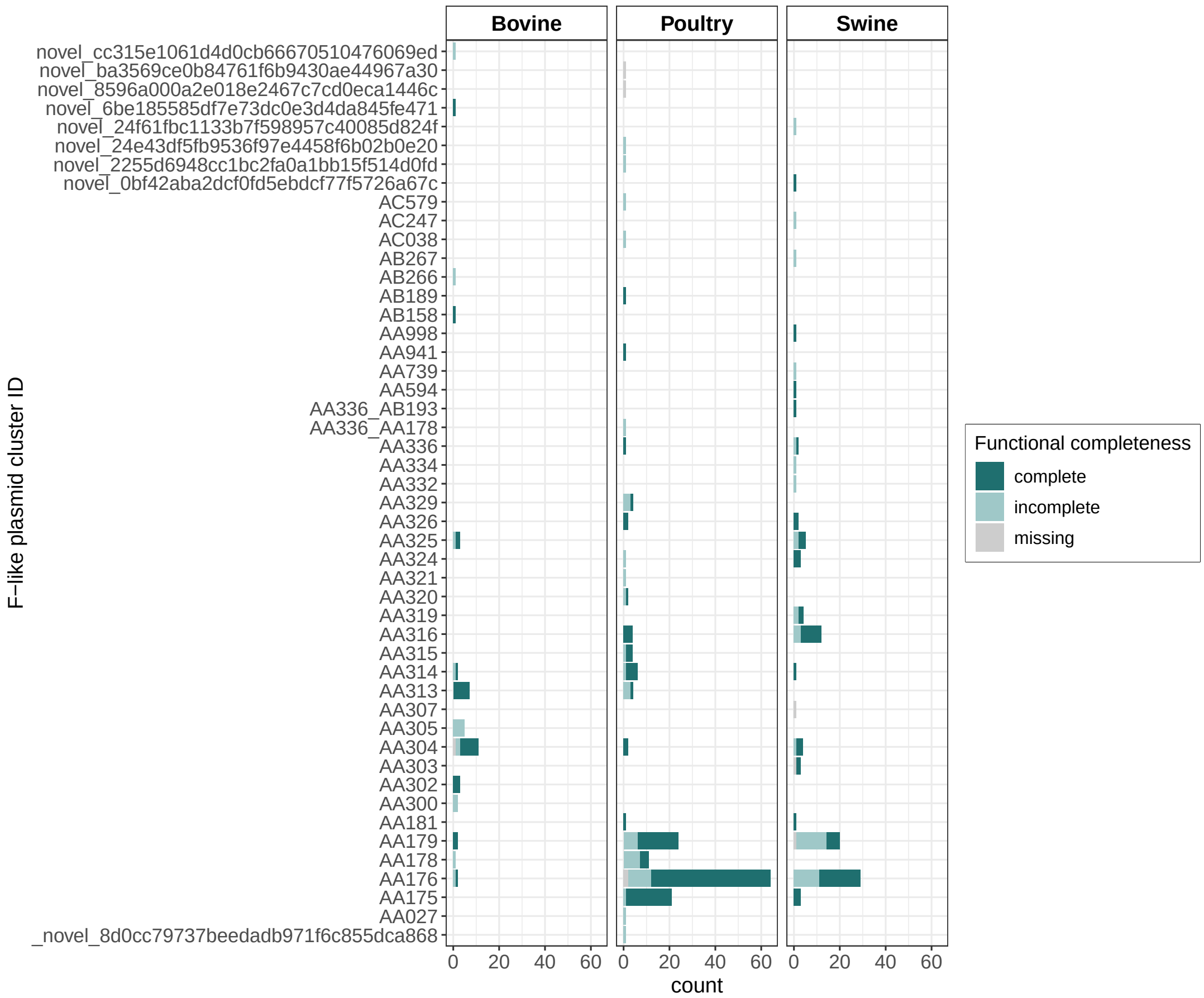

Figure S6: Distribution of F-like plasmid clusters in each animal environments. Bars indicate the proportion of each plasmid cluster classified as having a functionally complete F-like transfer operon (dark blue), incomplete F-like transfer operon (light blue) or missing an F-like transfer operon (grey). Plasmid clusters were assigned using MOB-Brecon from the MOBSuite package (Supplementary Text).

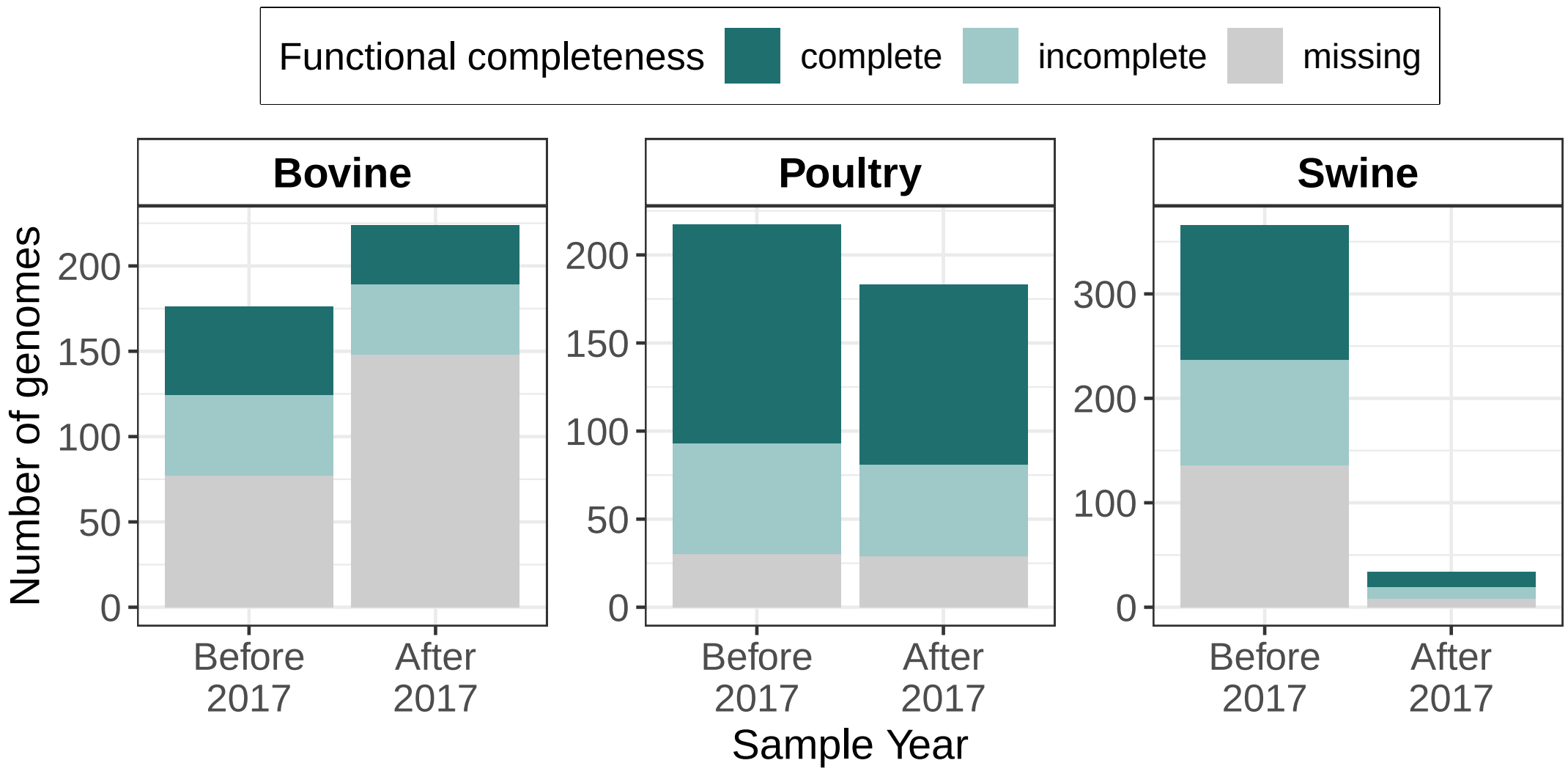

Figure S7: Proportion of *E. coli* isolates in each animal environment with complete, incomplete and missing F-like transfer operons sampled before 2017 and after 2017 (which also includes 2017). We find no difference in F-like transfer operon functional completeness in isolates sampled before and after 2017 ($\chi^2(1) = 3.63, p = 0.057$, Cramér's V effect size $= 0.05$ $[0.00, 0.11]$)

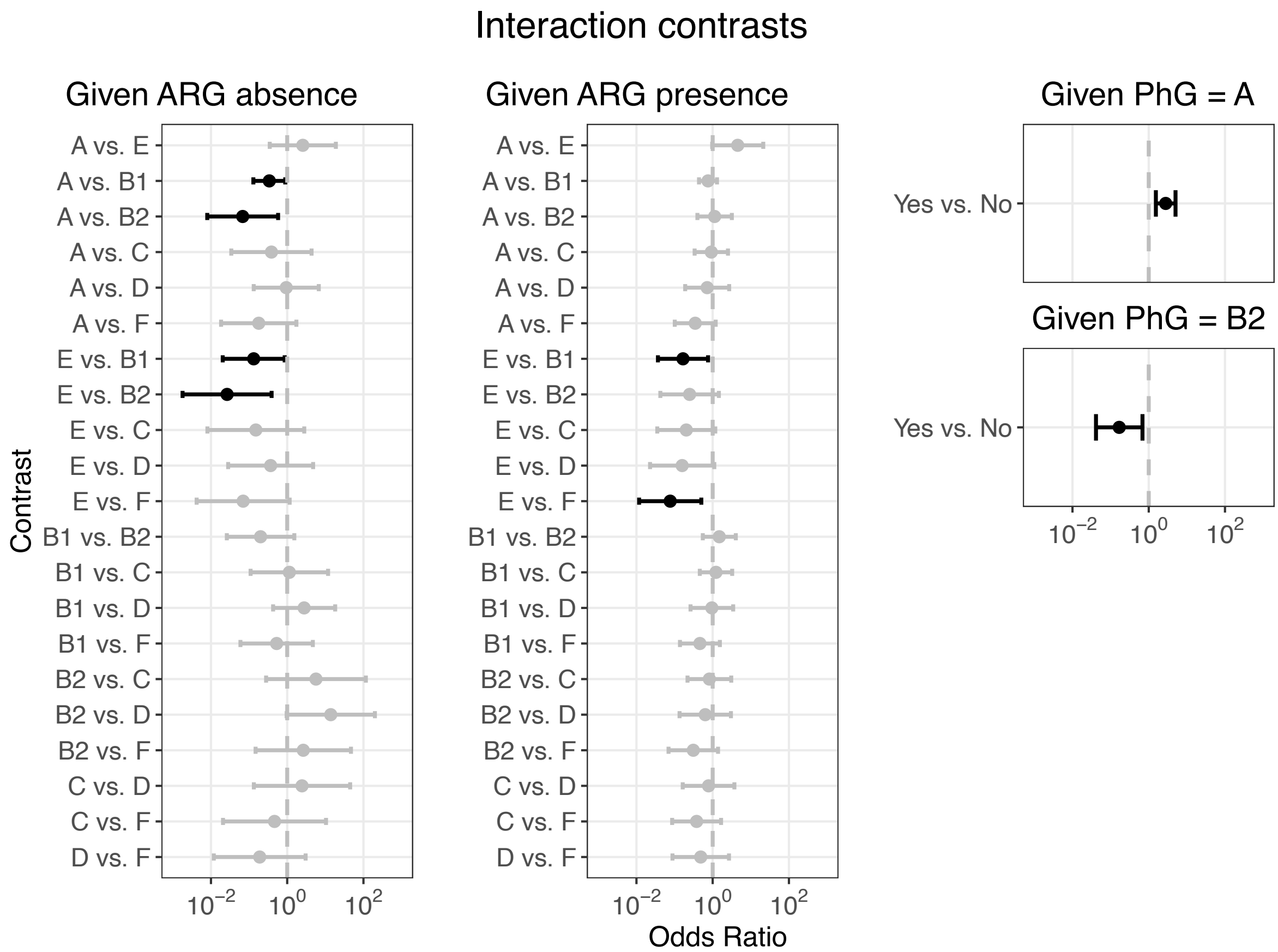

Figure S8: Contrasts for the interaction term between antibiotic resistance gene (ARG) presence and *E. coli* phylogroup (PhG). We report odds ratio of harboring a complete F-like plasmid in one group vs the other conditioned on a specific variable. The x-axis is on a log scale, and error bars indicate 95% confidence limits. Contrasts that were significant ($p < 0.05$) after correction for multiple testing are shown in black, while nonsignificant contrasts are grey.

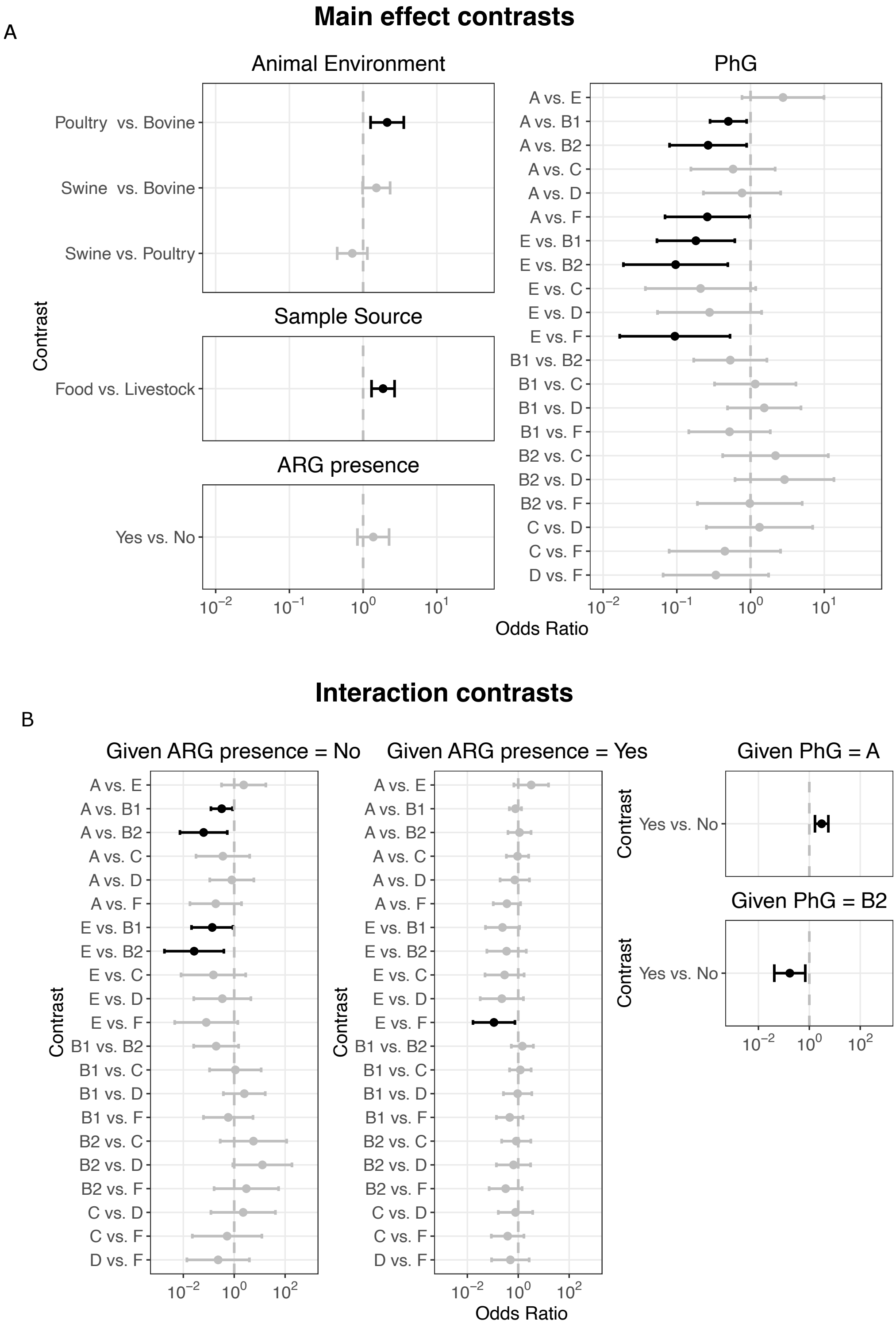

Figure S9: Logistic regression analyses using a reduced dataset of 1160 genomes. The reduced dataset was obtained after removing highly similar genomes ($\geq$99.99% ANI) with similar plasmid content, which were sampled at the same location and time. The model selection procedure was repeated for this dataset and the same covariates were selected. **A.** Main effect contrasts of harboring a complete F-like plasmid in one group vs. the other after adjusting for all other covariates. **B.** Contrasts for the interaction term between antibiotic resistance gene (ARG) presence and *E. coli* phylogroup (PhG). Odds ratios of harboring a complete F-like plasmid in one group vs the other conditioned on a specific variable are reported. For both A and B, The x-axis is on a log scale, and error bars indicate 95% confidence limits. Contrasts that were significant ($p < 0.05$) after correction for multiple testing are depicted in black.

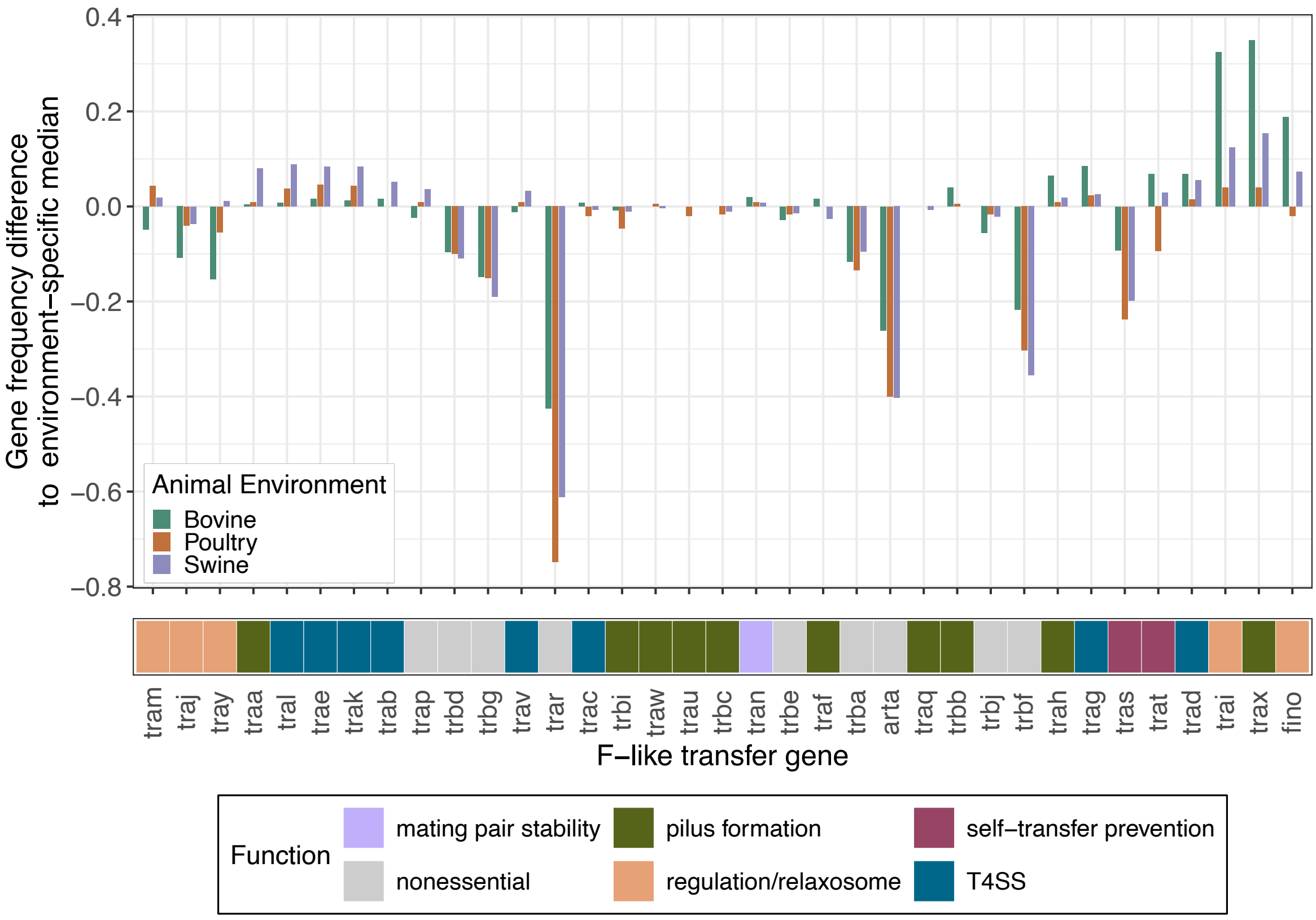

Figure S10: The difference between the frequency of each F-like transfer gene and the median gene frequency of the entire operon within each animal environment: bovine (green), poultry (orange), and swine (purple). Transfer genes are colored by their role in conjugation.

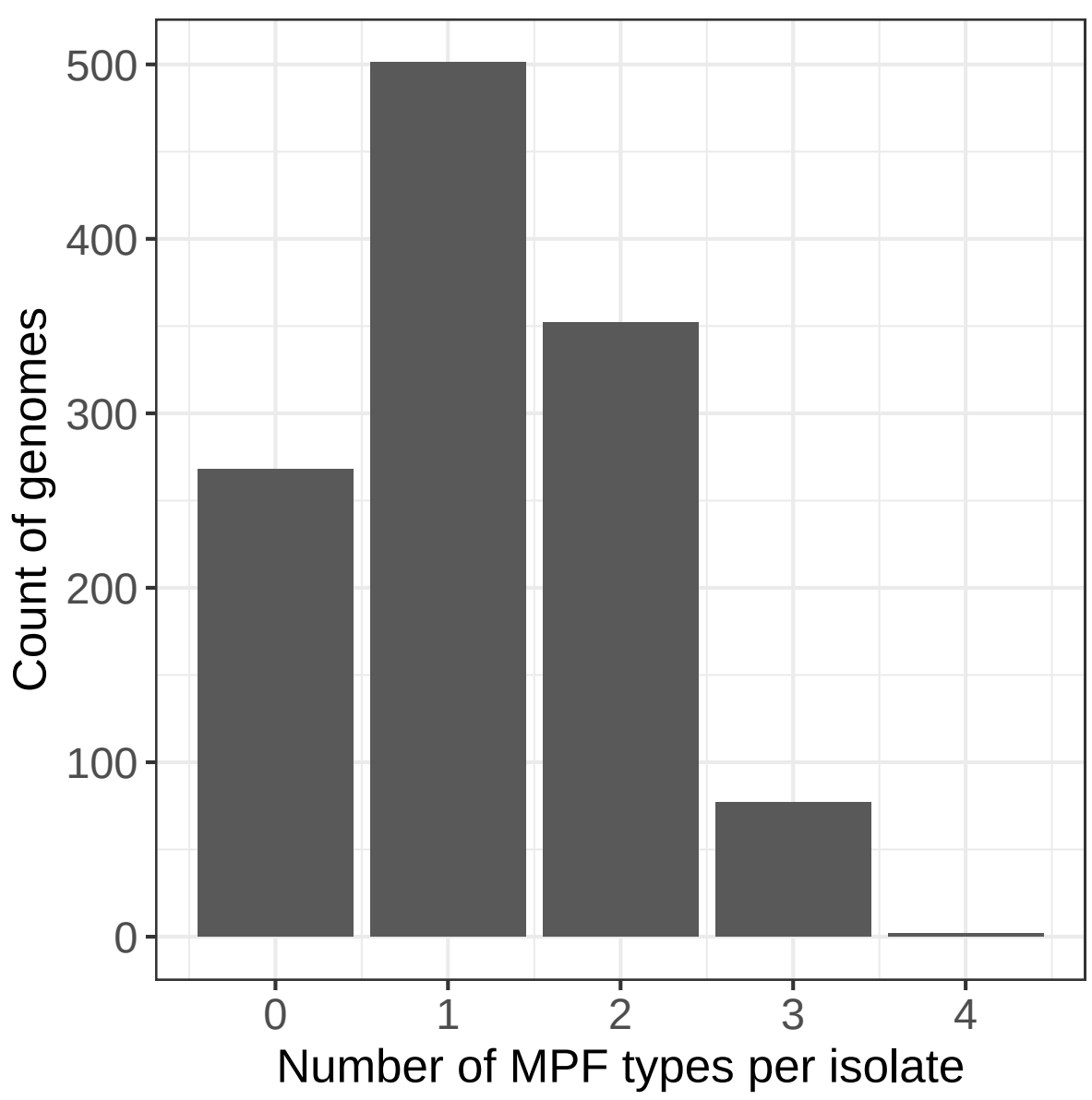

Figure S11: Distribution of the number of MPF types detected per isolate using geNomad.

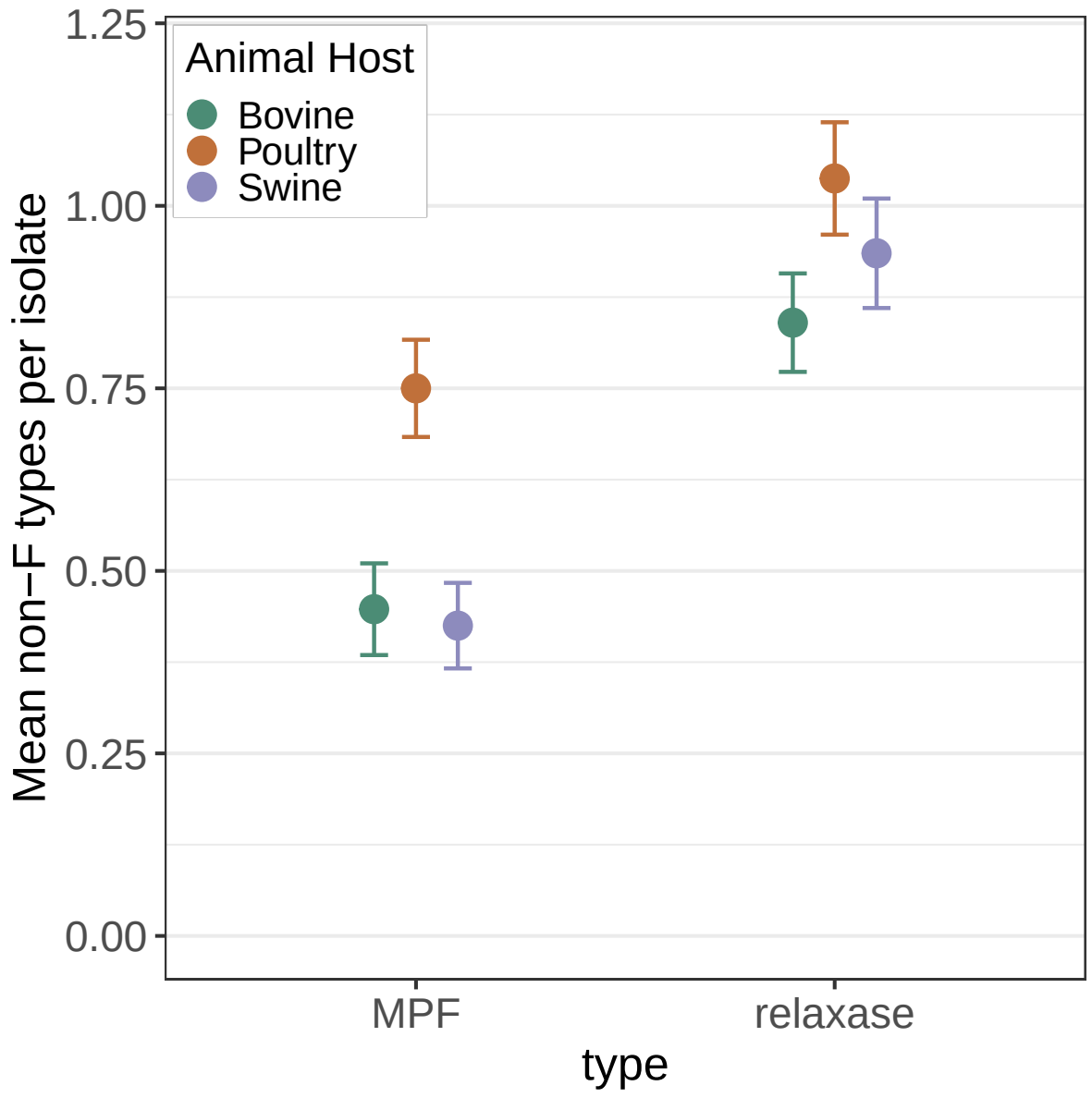

Figure S12: Mean number of non-F MPF types and relaxase types per isolate in each animal environment. MPF and relaxase types were detected using geNomad. Error bars indicate 95% confidence intervals of the mean.

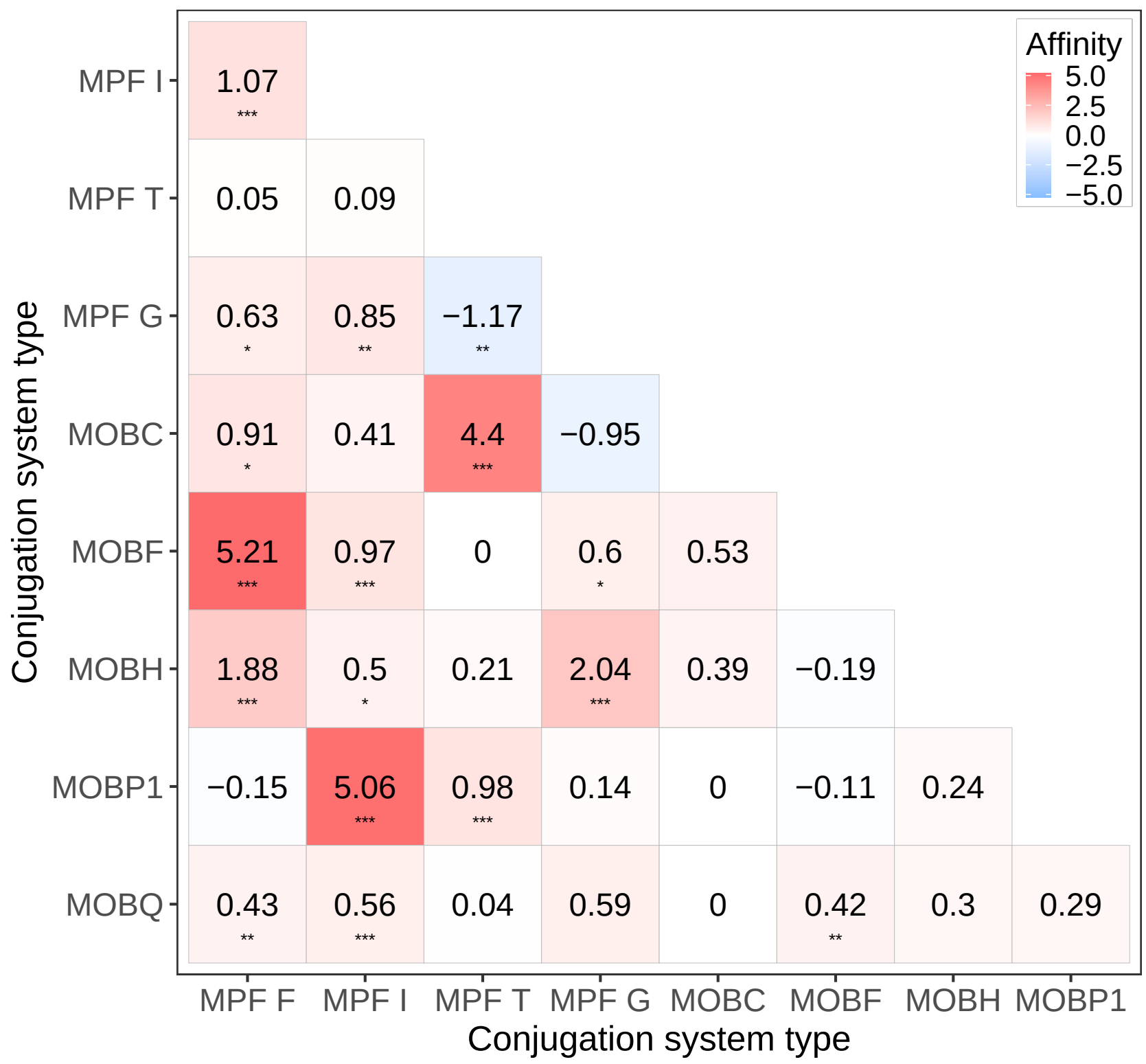

Figure S13: Affinity matrix describing the odds of finding pairs of conjugation system and relaxase types together in a isolate. Positive values (red) indicate higher odds of finding a pair together than the null expectation of random assortment. Negative values (blue) indicate higher odds of finding a pair in two different isolates than the null expectation. Zero values (white) indicates co-occurrence as expected based on individual frequencies (null expectation). Significance levels: *p $\leq$ 0.05, **p $\leq$ 0.01, ***p $\leq$ 0.001.

S2 Supplementary Tables

F-like tra genes	Function	n reference sequences	Min protein length	Median protein length	Max protein length
*traM**	regulation/relaxosome	27	123	127	168
*traJ**	regulation/relaxosome	32	67	228	525
*traY**	regulation/relaxosome	22	60	75	133
*traA**	pilus formation	25	79	120	132
*traL**	T4SS	13	98	103	104
*traE**	T4SS	24	121	188	188
*traK**	T4SS	24	169	242	475
*traB**	T4SS	26	422	475	866
traP	nonessential	27	131	196	208
trbD	nonessential	27	63	106	136
trbG	nonessential	9	77	83	88
*traV**	T4SS	27	171	171	204
*traR**	nonessential	13	57	73	74
*traC**	T4SS	33	785	876	882
*trbI**	pilus formation	24	101	128	154
*traW**	pilus formation	34	195	210	248
*traU**	pilus formation	23	322	330	364
trbC	pilus formation	36	200	212	221
*traN**	mating pair stability	36	284	602	651
trbE	nonessential	21	71	85	86
*traF**	pilus formation	32	247	249.5	331
trbA	nonessential	17	112	115	115
artA	nonessential	5	104	104	111
*traQ**	pilus formation	21	78	94	97
*trbB**	pilus formation	24	160	181	203
trbJ	nonessential	24	89	104	147
trbF	nonessential	24	37	130	147
*traH**	pilus formation	26	426	457.5	460
*traG**	T4SS	35	268	940	984
traS	self-transfer prevention	15	60	169	182
traT	self-transfer prevention	35	220	243	293
*traD**	T4SS	47	46	732	1767
*traI**	regulation/relaxosome	39	130	1756	1766
*traX**	pilus formation	39	153	248	405
finO	regulation/relaxosome	31	67	186	211

Table S1: F-like transfer gene markers, their functions and the number of reference genes used for transfer operon detection. Functional roles have been adapted from [6]. Essential genes used to classifying functional completeness of the F-like transfer operon are indicated with a *.

**Animal Environment**	**Complete**		**Incomplete**		**Missing**	
	Residual	p	Residual	p	Residual	p
Bovine	-8.24	$< .001$	-2.37	0.162	10.53	$< .001$
Poultry	9.29	$< .001$	1.39	1.000	-10.70	$< .001$
Swine	-1.05	1.000	0.97	1.000	0.17	1.000

Table S2: Post-hoc comparisons of the association between F-like transfer operon completeness and animal environment using using Pearson's Chi-squared Test. Positive values indicate greater counts than expected in that category, negative values indicate fewer counts than expected in that category. We report adjusted standardized Pearson residuals and Bonferroni-corrected *p* values.

Variable 1	Variable 2	X^2	df	p-value	Cramér's V [95% CI]
Animal Environment	PhG	316.39	12	$p < .001$	0.36 [0.31, 0.39]
Animal Environment	Sample Source	485.66	2	$p < .001$	0.64 [0.58, 0.69]
Animal Environment	ARG presence	96.94	2	$p < .001$	0.28 [0.22, 0.34]
PhG	Sample Source	53.88	6	$p < .001$	0.20 [0.12, 0.25]
PhG	ARG presence	42.93	6	$p < .001$	0.18 [0.10, 0.23]
Sample Source	ARG presence	3.37	1	$p = .066$	0.04 [0.00, 0.11]

Table S3: Associations between Animal environment, PhG, Sample Source, and ARG presence tested with Chi-squared Test of Independence. Effect sizes for associations was estimated using Cramér's V and 95% confidence intervals are reported.

Model Formula	Residual df	Residual Deviance	AIC
**Animal Environment + PhG + Sample Source + ARG Presence**	**1189**	**1433**	**1455**
Animal Environment + PhG + Sample Source	1190	1439	1459
Animal Environment + PhG + Sample Source + Sampling Year	1189	1438	1460
Animal Environment + PhG + ARG Presence	1190	1441	1461
Animal Environment + PhG + ARG Presence + Sampling Year	1189	1439	1461
Animal Environment + PhG	1191	1445	1463
Animal Environment + PhG + Sampling Year	1190	1444	1464
PhG + Sample Source + ARG Presence	1191	1452	1470
PhG + Sample Source + ARG Presence + Sampling Year	1190	1450	1470
PhG + Sample Source + Sampling Year	1191	1464	1482
PhG + Sample Source	1192	1466	1482
Animal Environment + Sample Source + ARG Presence + Sampling Year	1194	1476	1488
Animal Environment + Sample Source + ARG Presence	1195	1480	1490
Animal Environment + ARG Presence + Sampling Year	1195	1480	1490
Animal Environment + ARG Presence	1196	1483	1491
Animal Environment + Sample Source + Sampling Year	1195	1484	1494
Animal Environment + Sampling Year	1196	1486	1494
Animal Environment + Sample Source	1196	1487	1495
Animal Environment	1197	1489	1495
PhG + ARG Presence	1192	1501	1517
PhG + ARG Presence + Sampling Year	1191	1501	1519
Phylogenetic Group (PhG)	1193	1510	1524
Sample Source + ARG Presence + Sampling Year	1196	1517	1525
PhG + Sampling Year	1192	1510	1526
Sample Source + ARG Presence	1197	1524	1530
Sample Source + Sampling Year	1197	1537	1543
Sample Source	1198	1545	1549
ARG Presence + Sampling Year	1197	1575	1581
ARG Presence	1198	1578	1582
Sampling Year	1198	1591	1595

Table S4: Selection of main-effects logistic regression model to predict the presence of a complete F-like operon in a genome. All combinations of explanatory variables (Animal Environment, Phylogroup (PhG), Sample Source and ARG presence and Sampling Year) were tested. Models are compared using Akaike Information Criterion (AIC). Lower values of AIC indicate better model fit. The model that includes all Animal Environment, Phylogroup (PhG), Sample Source and ARG presence had the lowest AIC (in bold).

**Interaction Term**	**df**	**Deviance**	**Residual df**	**Residual Deviance**	**AIC**	***p*-value**
Animal Environment × PhG	12	18.4	1177	1414.6	1460	0.10
Animal Environment × ARG Presence	2	3.77	1187	1429.3	1455	0.15
Animal Environment × Sample Source	2	5.34	1187	1427.4	1453	0.069·
**PhG × ARG Presence**	**6**	**18.7**	**1183**	**1414.1**	**1448**	**0.0047****
PhG × Sample Source	6	11.8	1183	1421.5	1455	0.067·
Sample Source × ARG Presence	1	1.05	1188	1432.0	1456	0.30

Table S5: Likelihood ratio tests for interaction terms added to the main effects model containing Animal Environment, PhG, Sample Source and ARG Presence as covariates. The interaction term between PhG and ARG presence significantly improved model fit and was added to the main effects model. This model had the lowest AIC among models tested and was used for subsequent analyses.** $p < 0.01$; * $p < 0.05$; **.** $p < 0.1$.

S3 Supplementary Text

S3.1 Genome metadata

The metadata for the genomes were retrieved from [7] where extensive metadata standardization was done to ensure its quality. We describe here briefly the details of the specific metadata variables used in this paper:

- Animal Host: which animal production group the sample is associated with (Poultry, Swine or Bovine)
- Phylogenetic group: the phylogenetic group (PhG) was determined *in silico* using ClermonTyping (version 1.0.0 [8]) and MLST performed with mlst (version 2.16.2 [9, 10]).
- ARG presence: Binary value indicating whether the sample contained antimicrobial resistance genes (ARGs). ARGs were screened using ABRicate (version 0.8.13 [11]) with the ResFinder database (version of 22 April 2019 [12]). If the assembly contained ARG(s), ARG presence was 1.
- Sample Source: Indicates whether the samples were recovered from food products (Food) or from the animals themselves (Livestock). We assume, throughout most of this paper, that the food isolates form part of the broader ecology of the animal environment itself. This is because *E. coli* recovered from food products can be introduced from various sources at multiple stages of the animal production production process [13]. For instance, extra-intestinal pathogenic *Escherichia coli* (ExPEC) commonly found on poultry food samples have been shown to likely stem from the live bird itself rather than from humans [14].
- Sampling Year: Indicates whether the sampling year was before 2017 or 2017 and later (63.2% of isolates).

S3.2 Reference *tra* gene database construction

We curated a non-redundant reference dataset of amino acid sequence homologues of 35 F-like *tra* genes comprising 917 sequences in total (Supplementary Table S1). The dataset was adapted from Fernandez-Lopez *et al* [15] by removing duplicates, re-annotating the sequences based on updated NCBI protein accession numbers and manually adding missing annotations. The reference *tra* sequences stem from 70 F-like plasmids found in Enterobacteriaceae: *E. coli* (656 *tra* sequences from 48 plasmids), *Salmonella enteric*a (164 *tra* sequences from 14 plasmids), *Salmonella sp.* (24 sequences from 1 plasmid), *Shigella dysenteriae* (2 sequences from 1 plasmid), *Shigella flexneri* (37 sequences from 4 plasmids) , *Shigella sonnei*(4 sequences from 1 plasmid) and 33 sequences with an unidentified host.

S3.3 F-like transfer gene essentiality

In total we detected 35 genes of the F-like transfer operon, of which 26 have a characterized function in conjugation [6, 16, 17]. From this set, 23 can be described as ”essential” for self-transfer (Table S1), i.e. the gene has a demonstrated function in

conjugation or its regulation, and mutations in these genes block or reduce plasmid transfer [6, 16, 17]. Entry and surface exclusion genes *traS* and *traT* are not considered essential genes, because they are not involved in the core conjugation machinery and experimental evidence shows that deleting these genes results in increased transfer rates [18, 19]. The fertility inhibition gene *finO* is also not essential for conjugation, as it negatively regulates *tra* gene expression, with higher rates of transfer observed in the classic F plasmid which has a non-functional *finO* gene [6, 20].

S3.4 Mobility typing scheme from Coluzzi *et al.*

We performed mobility typing on each genome using the classification scheme from Coluzzi *et al* [5]. This mobility typing scheme uses the presence of the relaxase and a small number of other conjugation-related genes (including VirB4, T4CP and MPF genes) to classify a plasmid as conjugative (conj; containing the relaxase, VirB4, T4CP and at least 3 MPF proteins), mobilizable (mob; containing the relaxase but missing other components required to be conjugative), non-mobilizable (mobless; lacking relaxases) or partially decayed conjugative (pdconj; a subcategory of MOB plasmids with more than 5 conjugation proteins excluding the relaxase). Again, we used the set of F-like transfer genes we identified in each genome for this classification.

S3.5 Plasmid reconstruction and clustering

We used MOB-recon from MOB-Suite (v.3.1.9, default parameters, using a set of closed *Enterobacteriaceae* genomes for chromosome depletion) for plasmid reconstruction and clustering. The default mash distance threshold of 0.05 was used to assign plasmid cluster identities. Each genome is represented as a binary vector that describes the presence and absence of these plasmid cluster IDs.

The plasmid clusters specifically associated with the F-like plasmid-associated contigs identified using our transfer gene finding protocol were considered to be F-like plasmid clusters. Only 38.5% of the genomes containing an F-like transfer operon (n=786) could be mapped to an F-like plasmid cluster.

S3.6 Identifying redundant genomes for sensitivity analyses

Pairwise average nucleotide identity (ANI) was computed using default settings of fastANI (v.1.34) [21]. Out of all the genomes sampled at the same time and location, we found 35 clusters of genomes (101 genomes in total) with pairwise ANI $\geq$99.99% , which is a recommended ANI threshold for strain classification [3]. Out of these clusters, 20 (60 genomes in total) also had similar plasmid content, where pairwise plasmid content similarity was measured using the Jaccard similarity index on the presence/absence vector of plasmid cluster identities (see Section S3.5). Therefore, 40 out of 1200 genomes (0.03% of the total dataset) are presumably redundant. We kept a random representative genome from each of these clusters to form a reduced dataset of 1160 genomes to test the sensitivity of our logistic regression results to redundant genomes.

S3.7 Co-occurrence of conjugation types

Co-occurrence between two conjugation types in an isolate was assessed using a prevalence-insensitive metric called affinity [22]. For a pair of conjugation types A and B, affinity measures the odds of finding type B in an isolate given that the isolate already has type A. A positive affinity (> 0) indicates that types A and B co-occur more frequently than expected under the null distribution, whereas a negative value (< 0) indicates that they occur in separate genomes more frequently than expected.

Pairwise affinities were computed for all pairs of conjugation types, which includes 6 pairs of MPF types, 10 pairs of relaxase types and 20 combinations of relaxase and MPF types using the R package CooccurrenceAffinity v.1.0.2 [23]. Since we found only 1 occurrence of MOB V in the entire set of genomes, we excluded this type from the analyses.

References

1. Chklovski, A., Parks, D. H., Woodcroft, B. J. & Tyson, G. W. CheckM2: a rapid, scalable and accurate tool for assessing microbial genome quality using machine learning. en. *Nature Methods* **20,** 1203–1212 (Aug. 2023).
2. Parks, D. H., Imelfort, M., Skennerton, C. T., Hugenholtz, P. & Tyson, G. W. CheckM: assessing the quality of microbial genomes recovered from isolates, single cells, and metagenomes. *Genome Research* **25,** 1043–1055 (July 2015).
3. Viver, T. *et al.* Towards estimating the number of strains that make up a natural bacterial population. en. *Nature Communications* **15,** 544 (Jan. 2024).
4. Rodriguez-R, L. M. *et al.* An ANI gap within bacterial species that advances the definitions of intra-species units. *mBio* **15,** e02696–23 (Dec. 2023).
5. Coluzzi, C., Garcillán-Barcia, M. P., de la Cruz, F. & Rocha, E. P. Evolution of Plasmid Mobility: Origin and Fate of Conjugative and Nonconjugative Plasmids. *Molecular Biology and Evolution* **39,** msac115 (June 2022).
6. Koraimann, G. Spread and Persistence of Virulence and Antibiotic Resistance Genes: A Ride on the F Plasmid Conjugation Module. *EcoSal Plus* **8.** Publisher: American Society for Microbiology (July 2018).
7. Pires, J., Huisman, J. S., Bonhoeffer, S. & Van Boeckel, T. P. Increase in antimicrobial resistance in Escherichia coli in food animals between 1980 and 2018 assessed using genomes from public databases. *Journal of Antimicrobial Chemotherapy* **77,** 646–655 (Mar. 2022).
8. Beghain, J., Bridier-Nahmias, A., Nagard, H. L., Denamur, E. & Clermont, O. ClermonTyping: an easy-to-use and accurate in silico method for Escherichia genus strain phylotyping. en. *Microbial Genomics* **4,** e000192 (July 2018).
9. Jolley, K. A., Bray, J. E. & Maiden, M. C. J. Open-access bacterial population genomics: BIGSdb software, the PubMLST.org website and their applications. eng. *Wellcome Open Research* **3,** 124 (2018).
10. Seemann, T. *tseemann/mlst* original-date: 2014-05-03T09:12:11Z. July 2026.
11. Seemann, T. *tseemann/abricate* original-date: 2014-07-17T00:59:35Z. July 2026.
12. Bortolaia, V. *et al.* ResFinder 4.0 for predictions of phenotypes from genotypes. eng. *The Journal of Antimicrobial Chemotherapy* **75,** 3491–3500 (Dec. 2020).

13. Risalvato, J., Sewid, A. H., Eda, S., Gerhold, R. W. & Wu, J. J. Strategic Detection of Escherichia coli in the Poultry Industry: Food Safety Challenges, One Health Approaches, and Advances in Biosensor Technologies. en. *Biosensors* **15,** 419 (July 2025).
14. Bergeron, C. R. *et al.* Chicken as Reservoir for Extraintestinal Pathogenic Escherichia coli in Humans, Canada - Volume 18, Number 3—March 2012 - Emerging Infectious Diseases journal - CDC. en-us.
15. Fernandez-Lopez, R., de Toro, M., Moncalian, G., Garcillan-Barcia, M. P. & de la Cruz, F. Comparative Genomics of the Conjugation Region of F-like Plasmids: Five Shades of F. *Frontiers in Molecular Biosciences* **3** (2016).
16. Frost, L. S., Ippen-Ihler, K. & Skurray, R. A. Analysis of the sequence and gene products of the transfer region of the F sex factor. *Microbiological Reviews* **58.** Publisher: American Society for Microbiology, 162–210 (June 1994).
17. Firth, N., Ippen-Ihler, K., Skurray, R. A., *et al.* Structure and function of the F factor and mechanism of conjugation. *Escherichia coli and Salmonella: cellular and molecular biology, 2nd ed. ASM Press, Washington, DC,* 2377–2401 (1996).
18. Achtman, M., Kennedy, N. & Skurray, R. Cell–cell interactions in conjugating Escherichia coli: role of traT protein in surface exclusion. en. *Proceedings of the National Academy of Sciences* **74,** 5104–5108 (Nov. 1977).
19. Couturier, A., Fraikin, N. & Lesterlin, C. Exclusion systems preserve host cell homeostasis and fitness, ensuring successful dissemination of conjugative plasmids and associated resistance genes. *Nucleic Acids Research* **53,** gkaf898 (Sept. 2025).
20. Zatyka, M. & Thomas, C. M. Control of genes for conjugative transfer of plasmids and other mobile elements. *FEMS Microbiology Reviews* **21,** 291–319 (Feb. 1998).
21. Jain, C., Rodriguez-R, L. M., Phillippy, A. M., Konstantinidis, K. T. & Aluru, S. High throughput ANI analysis of 90K prokaryotic genomes reveals clear species boundaries. en. *Nature Communications* **9,** 5114 (Nov. 2018).
22. Mainali, K. P., Slud, E., Singer, M. C. & Fagan, W. F. A better index for analysis of co-occurrence and similarity. *Science Advances* **8.** Publisher: American Association for the Advancement of Science, eabj9204 (Jan. 2022).
23. Mainali, K. P. & Slud, E. CooccurrenceAffinity: An R package for computing a novel metric of affinity in co-occurrence data that corrects for pervasive errors in traditional indices. en. *PLOS ONE* **20.** Publisher: Public Library of Science, e0316650 (Jan. 2025).
